# Improving adherence to physical activity in treatment-resistant depression: Protocol for a pilot randomized controlled trial of a remotely delivered program

**DOI:** 10.1371/journal.pone.0330848

**Published:** 2025-09-02

**Authors:** Vanessa K. Tassone, Danika A. Quesnel, Karisa Parkington, Alice Rueda, Josh Martin, Gyu Hee Lee, Alyssa Teixeira, Melissa L. deJonge, Wendy Lou, David Wiljer, Benoit H. Mulsant, Catherine M. Sabiston, Venkat Bhat

**Affiliations:** 1 Interventional Psychiatry Program, St. Michael’s Hospital, Toronto, Ontario, Canada; 2 Institute of Medical Science, Temerty Faculty of Medicine, University of Toronto, Toronto, Ontario, Canada; 3 Faculty of Kinesiology and Physical Education, University of Toronto, Toronto, Ontario, Canada; 4 Department of Psychological Clinical Sciences, University of Toronto Scarborough, Toronto, Ontario, Canada; 5 Department of Biostatistics, Dalla Lana School of Public Health, University of Toronto, Toronto, Ontario, Canada; 6 Institute of Health Policy, Management and Evaluation, Dalla Lana School of Public Health, University of Toronto, Ontario, Canada; 7 University Health Network, Toronto, Ontario, Canada; 8 Department of Psychiatry, University of Toronto, Toronto, Ontario, Canada; 9 Centre for Addiction and Mental Health, Toronto, Ontario, Canada; 10 Mental Health and Addictions Services, St. Michael’s Hospital, Toronto, Ontario, Canada; Tehran University of Medical Sciences, IRAN, ISLAMIC REPUBLIC OF

## Abstract

**Background:**

At least 30% of individuals with major depressive disorder do not respond to conventional treatments (i.e., they meet the criteria for treatment-resistant depression [TRD]). Alternative therapeutic modalities are needed. Some studies have reported that physical activity (PA) programs can improve depressed mood and reduce depressive symptoms. However, few studies to date have examined the effects of PA as an adjunct to standard treatment for TRD. The MoveU.HappyU PA program has been shown to improve depressive symptoms in university students. Before a definitive trial testing MoveU.HappyU in TRD can be designed, pilot data is needed.

**Methods:**

The current study is a single-site, pilot, two-arm randomized controlled trial. It will investigate the feasibility of randomizing 30 adult participants with TRD to: (1) a remotely delivered four-week MoveU.HappyU adjunct to treatment as usual (TAU), or (2) TAU. Acceptability of the PA program will also be assessed. Participants randomized to the PA program will meet weekly with a program trainer to engage in PA counselling and structured PA. They will also be instructed to independently complete 120 minutes of PA per week. The four-week intervention period will be followed by six weeks of observation. Throughout the study, both groups will receive the same digital monitoring via self-report questionnaires and a wearable device, as well as traditional monitoring (i.e., clinical assessments administered by a masked rater).

**Discussion:**

This pilot study will assess the feasibility of a trial implementing MoveU.HappyU for TRD and generate clinical parameter estimates for larger studies. This line of research highlights the importance of PA programs that integrate personalized PA with PA counselling. It will also influence the development of interventions that are more tailored and effective.

**Trial registration:**

ClinicalTrials.gov NCT06404320

## Introduction

Approximately 5% to 17% of individuals experience major depressive disorder (MDD) within their lifetime [1]. MDD is a complex and multifaceted condition that can manifest as a range of somatic symptoms, demonstrating an interplay between mental and physical health. Evidence suggests that there is a non-linear dose-response relationship between physical activity (PA) and mental health. The benefits of moderate-to-vigorous PA (MVPA) are apparent for up to 50 minutes per day, whereas longer durations are associated with diminished mental health [2]. Studies have also reported that PA can improve depressed mood [3] and reduce depressive symptoms [4].

Challenges surrounding appropriate treatment for MDD remain, with at least 30% of individuals not responding to conventional treatments (i.e., meeting criteria for treatment-resistant depression [TRD]) [5]. There is a need for alternative interventions and new approaches for the management of TRD are being investigated. PA interventions can bolster the impact of conventional treatments [6]. The Canadian Network for Mood and Anxiety Treatments (CANMAT) has deemed exercise, a subset of PA which is planned, structured, and repetitive with the goal of physical fitness [7], to have Level 1 evidence [8]. As such, CANMAT recommends exercise as a first-line monotherapy for mild-to-moderate MDD and as a second-line adjunctive therapy for moderate-to-severe MDD [8]. Considering the relationship between mental and physical well-being, therapeutic modalities such as structured programs of PA and exercise could be of benefit in TRD.

A systematic review conducted in the year 2023 determined that few original studies have examined the effects of PA as an adjunctive treatment for TRD [9]. This research has investigated 12-week programs of aerobic (walking) [10] and combined (i.e., aerobic and resistance) [11] exercise, and 8 months of strength training [12,13]. There is also evidence for PA interventions in non-remitted MDD and depression that was poorly responsive to antidepressant treatment, with studies comparing high versus low weekly energy expenditures during a 12-week aerobic exercise intervention [14–22] and exploring the effects of 10 weeks of predominantly weight-bearing exercise classes [23]. Collectively, the results of this research has highlighted that PA can improve outcomes of MDD that has been treated with antidepressants, including depressive symptoms [10,13,23], quality of life [12,18], sleep quality [17], and cognitive function [19].

Individuals with MDD often struggle to follow medical treatment recommendations [24]. In particular, adherence to PA interventions is suboptimal and remains poorly understood [25]. Difficulty with PA engagement has been recognized as a challenge in clinical trials for MDD (e.g., [26–28]) and it may explain its lack of widespread adoption. Barriers to adherence to PA include lack of motivation and energy, which are prevalent symptoms of MDD [29,30]. Moreover, there are distinct clinical characteristics of TRD compared to non-TRD [31]. Given these challenges, there is a need for research investigating how to improve engagement in PA interventions in individuals with TRD.

Research has explicitly called for the integration of supervision, personalization, and behaviour change counselling into PA interventions for mental health to optimize engagement and efficacy [32]. The studies described above have incorporated varying degrees of supervision (i.e., fully supervised [11,12,23] and a combination of supervised and unsupervised sessions [10,14]). They have also offered opportunities for personalization, such as allowing participants to choose where to perform unsupervised exercises [10] and prescribing doses of exercise to be carried out at self-selected intensities [14]. This research has also implemented strategies to evoke behaviour change, including skills training, developing individually tailored plans [14], setting prompts/cues, and leveraging existing social relationships [10]. However, longer (as opposed to shorter) overall durations of PA interventions might hinder adherence in TRD and, in turn, impact their effectiveness [33]. In this context, low-demand PA interventions (e.g., of shorter durations, with reduced frequency and individual flexibility) could introduce PA to individuals with TRD and facilitate transition to self-directed PA over time.

MoveU.HappyU [34] is an evidence-informed PA program developed for university students. It is grounded in self-determination theory [35] and delivers individually-tailored PA in conjunction with PA counselling during weekly, supervised sessions with a program trainer [36]. Participants are also given agency to choose the frequency of unsupervised PA sessions within a given week. MoveU.HappyU has been shown to improve depressive symptoms and was deemed acceptable in a sample of university students seeking mental health support [36]. The existing evidence supports delivery of MoveU.HappyU over six weeks [36], making the program shorter in duration than previously investigated PA interventions for TRD. This might contribute to improved adherence and effectiveness [33]. Recently, MoveU.HappyU has also been delivered virtually using video conferencing software. Remote participation reduces barriers to engagement in the program, such as travel demands and perceived effort. Given its design and opportunity for virtual delivery, MoveU.HappyU might be a viable low-demand, adjunctive PA intervention for TRD. However, before a definitive trial can be designed for participants with TRD, pilot data are needed to demonstrate feasibility and provide clinical parameter estimates to inform the sample size of a larger randomized trial.

The integration of digital data capture and qualitative measures into PA trials for TRD could better inform participation in PA, including adoption and maintenance. Wearable devices provide passive measures of PA and related biometrics in near real-time and have successfully been used in PA interventions to promote PA participation [37]. Research has also used ecological momentary assessments to capture within-subject variations in PA and mood during participants’ everyday lives [3]. Combining digital technologies (e.g., continuous wearable device use paired with frequent and remote symptom monitoring via web-based platforms) in PA clinical trials could uncover related changes in mental health symptoms as they occur in the real-world. For instance, a device-based measure of step count demonstrated a positive association with depressive symptom improvements following psychotherapy intervention in MDD [38]. More traditional semi-structured interviews can capture participants’ first-hand experiences of the intervention, including barriers and facilitators to participation, and provide individual or contextual factors to complement the data collected through digital tools. This might be of particular importance in TRD. For example, focus groups have suggested that positive experiences of an exercise program contributed to attendance in participants who were being monitored and treated for depression [39]. Moreover, reminders prior to each session of a yoga intervention, and peer support and written instruction for home practice, were facilitators to participation amongst individuals with depression [40]. Thus, including digital technologies and qualitative methods into PA clinical trials in TRD could inform whether such interventions are able to engage this clinical population in PA and the design of future interventions.

### Objectives

We outline the protocol for a pilot randomized controlled trial that will deliver a remote, one-on-one, individualized PA program (modified MoveU.HappyU) as an adjunctive intervention to adults with TRD who are receiving treatment as usual (TAU). The trial will use both digital and qualitative methods to inform study outcomes. The primary objective of this trial is to assess the feasibility of randomizing adult participants with TRD to (1) the PA program adjunct to TAU versus (2) TAU (control). A secondary objective will assess the acceptability of the PA program through semi-structured interviews and an exit survey. Exploratory objectives are to: estimate the efficacy of the PA program by assessing change in depressive symptoms, anxiety symptoms, and quality of life; evaluate the effect of the PA program on physiological biometrics collected using a wearable device; and explore the perceptions and experiences of PA in adults with TRD.

## Methods

### Study design and setting

**Fig 1:**
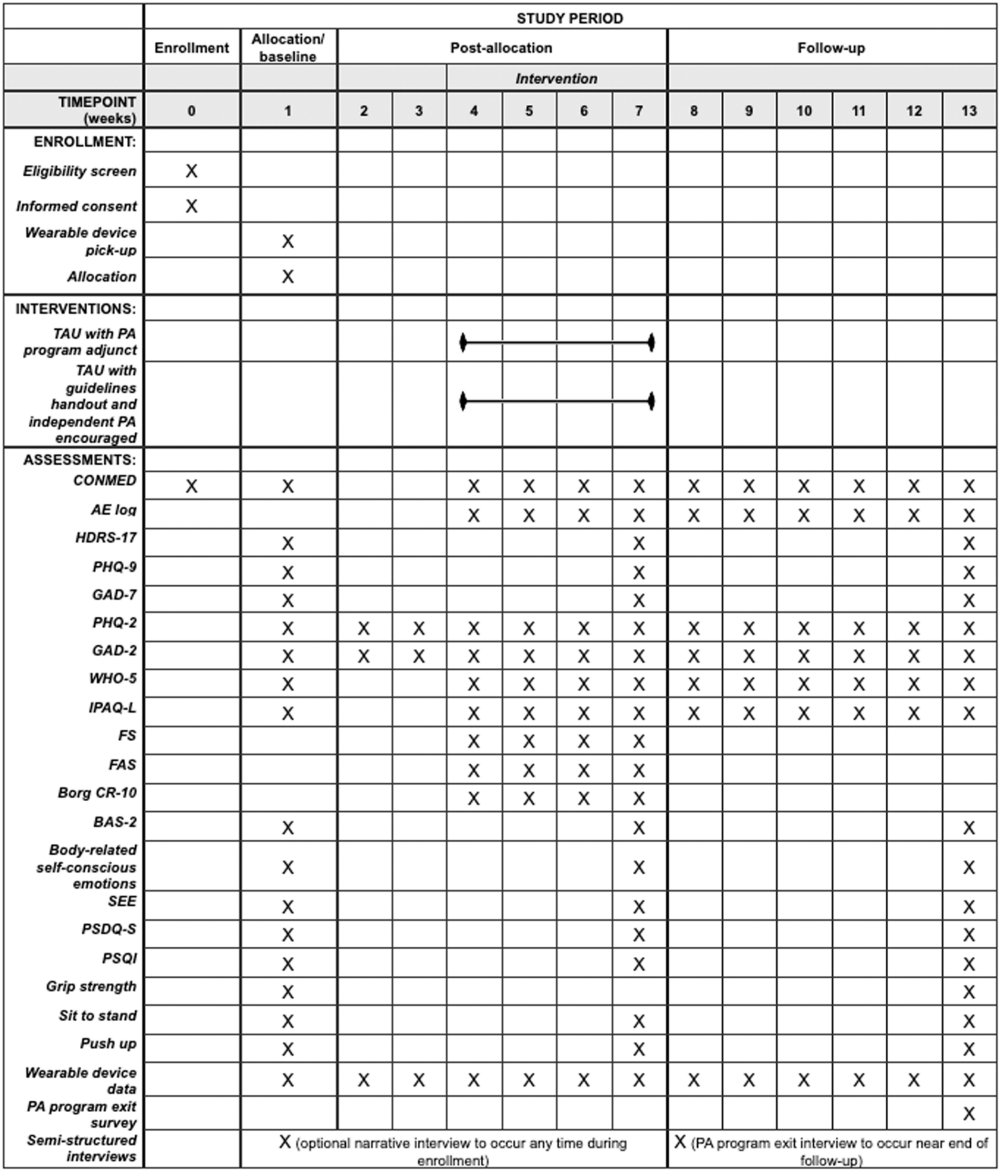
Schedule of enrollment, interventions, and assessments. Enrolled participants will be randomized to receive (1) MoveU.HappyU adjunct to TAU, or (2) TAU. Both groups will receive the same active and passive digital monitoring, as well as clinical assessments administered by a masked rater. Abbreviations: PA = physical activity, TAU = treatment as usual, CONMED = concomitant medication record, AE = adverse event, HDRS-17 = 17-item Hamilton Depression Rating Scale, PHQ-9 = Patient Health Questionnaire-9, GAD-7 = Generalized Anxiety Disorder-7, PHQ-2 = Patient Health Questionnaire-2, GAD-2 = Generalized Anxiety Disorder-2, WHO-5 = World Health Organization-Five Well-Being Index, IPAQ-L = International Physical Activity Questionnaire – Long Form, FS = Feeling Scale, FAS = Felt Arousal Scale, Borg CR-10 = Borg Rating of Perceived Exertion Category-Ratio scale, BAS-2 = Body Appreciation Scale-2, SEE = Self-Efficacy for Exercise Scale, PSDQ-S = Physical Self Description Questionnaire – Short Form (appearance, body fat, strength, activity, endurance, and global physical subscales), PSQI = Pittsburgh Sleep Quality Index.

**Fig 2:**
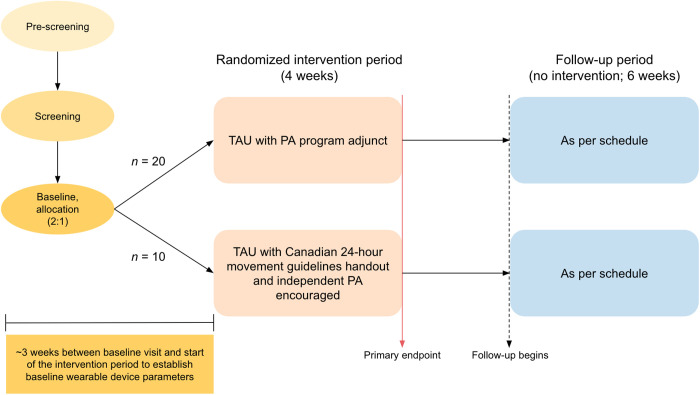
Study design. Participation in the study will span approximately 13 weeks. This will include a 3-week baseline period, 4-week intervention period, and 6-week follow-up period. Abbreviations: TAU = treatment as usual, PA = physical activity.

A single-site, pilot, randomized controlled trial with two parallel arms will be conducted at St. Michael’s Hospital (SMH), Unity Health Toronto (UHT), in Canada. The study team will be based at SMH-UHT and the University of Toronto. Participants with TRD will be randomized to one of two treatment arms using 2:1 allocation. Participants in the experimental group will continue TAU and receive a four-week PA program (MoveU.HappyU) adjunct. Participants in the control group will continue TAU without the PA program. They will instead be given a handout with the Canadian 24-hour movement guidelines [41] and encouraged to independently engage in PA. The schedule of enrollment, interventions, and assessments is outlined in Fig 1. The Standard Protocol Items: Recommendations for Interventional Trials (SPIRIT) and the Template for Intervention Description and Replication (TIDieR) checklists are available in S1 and S2 Files. Fig 2 presents the study design.

### Ethics approval and trial registration

This study was approved by the SMH-UHT Research Ethics Board (Approval No.: 23–069) on April 13, 2024. The full protocol is available in S3 File. The trial was registered on ClinicalTrials.gov on May 8, 2024 (identifier: NCT06404320).

This study will be conducted in compliance with the ethical principles outlined by the Research Ethics Board, the Tri-Council Policy Statement: Ethical Conduct for Research Involving Humans (2018), and the International Council of Harmonization – Good Clinical Practice E6. All participants will be required to provide informed, written consent to participate in the study.

The trial began recruitment on September 19, 2024 and is currently ongoing. The results of this trial will be published in peer-reviewed journals and presented at scientific conferences upon completion. Researchers interested in accessing de-identified participant data that is generated from this trial may contact the corresponding author.

### Eligibility criteria

To be considered for this study, potential participants must be capable of giving informed consent. Eligible participants will be sedentary adults (defined as engaging in less than 60 minutes [42] of MVPA per week) between the ages of 18 and 65 years, inclusive. Participants must meet diagnostic criteria for MDD without psychotic features according to the *Diagnostic and Statistical Manual of Mental Disorders – 5th Edition* [43] and be experiencing a major depressive episode (MDE), as confirmed by the Mini International Neuropsychiatric Interview (MINI) [44], at the time of enrollment. Participants must also have a Montgomery–Åsberg Depression Rating Scale (MADRS) [45] total score of ≥ 7 at screening (mild-to-severe MDE) after at least two trials of antidepressant therapy of adequate dose and duration during the current episode, as established by the Antidepressant Treatment History Form (ATHF) [46,47]. To be eligible, participants must be receiving treatments congruent with the CANMAT guidelines [48] with no changes in the 28 days before screening. The study team will also try to keep treatments unchanged during the four-week randomized intervention phase and the six-week follow-up phase.

Individuals will be excluded if they are experiencing symptoms of mania, hypomania, mixed episodes, or psychosis. They will also be excluded if they have received a diagnosis of an alcohol or substance use disorder within the past three months, as confirmed by the MINI [44]. Other psychiatric comorbidities will not be excluded. Individuals who are pregnant, have exercise-induced asthma, are at acute risk for a cardiovascular event (i.e., have had a cardiovascular event within the past 12 months), are taking medication that interferes with heart rate response to exercise (e.g., beta blockers), or have received intravenous ketamine treatment in the last two months will not be eligible for this study. Self-reported balance, gait, or locomotion difficulties that would preclude participation in a PA program, and any other medical contraindications according to the Physical Activity Readiness Questionnaire (PAR-Q) [49], are additional exclusion criteria. Individuals will also be excluded if they have any other condition that would adversely affect their ability to complete the study or its measures. Moreover, to facilitate use of the wearable device and completion of self-report questionnaires for digital symptom monitoring, individuals who do not have access to a smartphone and the Internet will be excluded. Individuals who do not speak English fluently enough to provide informed consent, understand study information, or answer questions accurately will not be eligible for this study.

### Sample size

Given the pilot nature of this trial, the anticipated effect size is undetermined. As such, power calculations were not conducted. Based on the 2:1 randomization design, a target sample of 30 participants (20 active, 10 control) with TRD will be sought [50]. The data generated from this trial will be used to estimate the variability of the efficacy outcomes and inform the sample size calculation for future larger scale trials.

### Recruitment

Participants will be recruited among patients seen at the Interventional Psychiatry Program (IPP) of SMH-UHT. Potential participants will be contacted to introduce the study to them. Social media advertisements and flyers posted at UHT-affiliated hospitals (i.e., SMH, St. Joseph’s Health Centre, or Providence Healthcare) will solicit self-referrals to the study. The IPP at SMH-UHT will be the sole study site.

### Randomization

A randomization schedule will be computer-generated using randomized blocks with a 2:1 ratio. Randomization will be stratified by sex, such that participants will be randomized within each sex category for balanced allocation across groups. Allocation concealment will be ensured using a computerized system (i.e., a secure randomization module within Research Electronic Data Capture [REDCap] [51]). The randomization module will only be accessible to an unmasked member of the study team who will not be involved in outcome assessment. Outcome assessors will be masked to group allocation.

Given that this is the first study to deliver MoveU.HappyU to individuals with TRD, a 2:1 allocation ratio producing a larger experimental group was selected to better inform acceptability of the PA program. Moreover, the limited literature investigating PA interventions as adjunctive treatment for TRD suggests that this might be a particularly difficult clinical population to recruit from. Thus, by maximizing the experimental group’s sample size, this pilot study also aims to address gaps in the literature about whether individuals with TRD are willing to be randomized to a PA program or a control group.

### Masking

While participants cannot be masked, the outcome assessor administering the primary depressive symptom measure (i.e., the 17-item Hamilton Depression Rating Scale (HDRS-17) [52]) will be masked to the allocation assignment. Participants will be told that the members of the research team (except for the program trainer) do not know which group they have been assigned to. Participants will also be instructed not to discuss details of their allocation when completing assessments. To evaluate the success of the masking at the end of the study, research staff administering the HDRS-17 [52] will complete a form in which they will guess which group the participant was randomized to [53].

### Intervention

#### Treatment as usual.

Prior to the intervention period, research staff will document all medications that are taken by participants on a regular basis and confirm that participants have not recently initiated treatment with psychotherapy, brain stimulation, or ketamine. Participants will continue their CANMAT-congruent [48] treatment for MDD throughout the study. Taking part in the study will not affect the standard of care, including any necessary changes to treatments. While the study protocol aims to avoid changes to participants’ treatment during the four-week randomized intervention phase and the six-week follow-up phase, changes that are clinically required will be allowed and recorded. According to the intention-to-treat principle, participants whose treatment is changed will remain in the trial.

#### Physical activity program (MoveU.HappyU).

In addition to continuing TAU, participants in the experimental group will engage in a remotely delivered (virtual) four-week adaptation of MoveU.HappyU and work individually with a program trainer for the duration of the intervention. The four-week duration aligns with that which has been used in a pragmatic randomized controlled trial in individuals being monitored or treated for depression [54].

**Fig 3:**
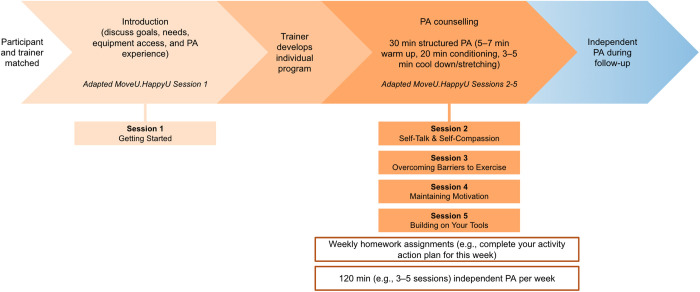
PA intervention. Participants will virtually meet one-on-one with their assigned program trainer for an introduction to the intervention. On a weekly basis during the intervention period, participants will receive one-on-one PA counselling using a version of the MoveU.HappyU Workbook that has been modified for TRD (e.g., content added to discuss the relationship between depression and PA, unhelpful thinking styles, steps for maintaining PA). During these sessions, participants will also engage in 30 minutes of supervised and structured PA that is tailored to their needs and goals. The intensity of these PA sessions will be self-selected and guided by the program trainer. The Talk Test [55] will be used to promote engagement in MVPA and monitor intensity. After each session with the trainer, participants will be asked to complete homework using the modified MoveU.HappyU Workbook and instructed to independently engage in PA for 120 minutes per week. The independent PA component can be completed by engaging in PA between three to five times in a given week. Abbreviations: PA = physical activity; TRD = treatment-resistant depression; MVPA = moderate-to-vigorous PA.

Program trainers will have completed a Bachelor’s degree in kinesiology, physical education, or sport science. Additionally, they will be required to have an accredited designation as an exercise professional (e.g., Personal Training Specialist [CanFitPro], Personal Trainer [International Sport Sciences Association], Certified Mental Performance Consultant [Canadian Sport Psychology Association], or Registered Kinesiologist). All program trainers will receive training for the MoveU.HappyU program, its modification for TRD (see Fig 3), and study-specific procedures. They will adhere to principles outlined in the intervention workbook, follow standardized operating procedures for the study, and engage in regular check-ins with the study coordinator to ensure consistency across participants. Program trainers and participants will be unmasked to group allocation.

MoveU.HappyU has been described in detail elsewhere [36] and its components are summarized in Fig 3. Its aim is to promote the ability to participate in self-directed PA [36]. For the current study, MoveU.HappyU was modified for individuals with TRD and delivered remotely. It was also shortened into a four-week program by combining sessions one and two of the original intervention (‘Getting Started’ and ‘Goal Setting’) to provide an overall introduction to the program. This introduction will occur during a three-week baseline period. Afterwards, the program trainer will develop an individualized and tailored PA program for the participant.

During the intervention period, participants will virtually meet with the program trainer once a week for four consecutive weeks. If a participant must reschedule a supervised PA session, it will be rescheduled to occur within the same week as originally scheduled. Briefly, these weekly sessions will consist of 30 minutes of PA counselling and 30 minutes of supervised and structured PA. PA counselling will involve the implementation of behaviour change strategies [56], including setting goals on a weekly basis, assessing progression toward goals, developing action plans, identifying barriers and enablers to achieving goals, and establishing cues or prompts for self-directed PA [36]. Although the structured PA program will differ across participants based on their interests and access to specific equipment (e.g., dumbbells), the overall organization of the weekly supervised PA sessions will be standardized across participants (i.e., warm up involving light-to-moderate intensity cardiorespiratory and endurance activities followed by aerobic or resistance exercises and cool down/stretching; Fig 3). Participants will also be instructed to independently engage in PA for 120 minutes per week outside of their session with the trainer at 50% to 65% of their maximum capacity. Though the independent sessions will be unsupervised, the participant’s total weekly duration of PA can be estimated using self-report and validated with data from the wearable device.

### Digital symptom monitoring

REDCap will be used to actively collect self-report data from all participants for the duration of their enrollment in the trial. On a daily basis, participants will complete brief two-item versions of the Patient Health Questionnaire (PHQ-2) [57] and Generalized Anxiety Disorder Scale (GAD-2) [58] that have been modified for daily administration, and a measure of the number of minutes of PA that the participant engaged in within the past day. The PHQ-2 [57] and GAD-2 [58] were adapted for daily administration by modifying the timeframe to “*over the past day*” and the response options to “*not at all*”, “*several times a day*”, “*more than half the day*”, and “*nearly all day*”. On a weekly basis during the intervention and follow-up periods, participants in both groups will independently complete the World Health Organization-Five Well-Being Index (WHO-5 [59]; modified for weekly administration) and the International Physical Activity Questionnaire – Long Form (IPAQ-L) [60].

### Wearable device

A wearable device, the Oura Ring (Oura Health Oy) [61], will be used to passively and continuously collect physiological biometrics that are relevant in the context of PA and mental health (e.g., the metabolic equivalent of task minutes, respiratory rate, blood oxygen saturation, heart rate variability, sleep metrics) from all participants. Participants will wear a fitted smart ring for the duration of the study and be asked to synchronize their data with a mobile application on a daily basis. Participants can also use the mobile application to monitor their own data.

### Semi-structured interviews

Virtual semi-structured exit interviews will be held with participants who were allocated to the experimental group to discuss the acceptability of the PA intervention. Exit interviews will explore perceptions of incorporating PA into treatment for TRD, factors affecting participation in the PA program (e.g., attendance to supervised sessions and engagement in independent PA), and maintenance of independent PA during the follow-up period. Beneficial aspects, as well as dislikes or barriers to participation in the program, will also be explored. Interviews will be conducted one-on-one with designated research staff. They will be scheduled near the end of the follow-up period and take approximately 45–60 minutes to complete. The interviews will be recorded for subsequent transcription and analysis.

### Study procedures

#### Pre-screening and consent.

Individuals who are interested in participating in the study will complete a pre-screening survey as a preliminary measure of their eligibility. Individuals who appear eligible after completing the survey will be sent the electronic informed consent form, demographics form, medical history, smoking history, and family history form, and PAR-Q [49] via REDCap. The consent form (version 5.0, dated January 30, 2025) is available in S4 File. If an individual agrees to participate in the study, they will digitally sign the consent form and be prompted to schedule their virtual screening visit for a comprehensive eligibility assessment. Participants may withdraw their consent at any time. Reasons for withdrawal will be documented as part of the study’s feasibility outcomes.

#### Screening.

The virtual screening visit will begin with participants reviewing the consent form with a member of the study team. Participants will also be offered the opportunity to participate in virtual semi-structured interviews as part of a narrative inquiry study to explore their experiences of depression and PA. The target sample size for these interviews will be seven to 10 participants. Their eligibility will subsequently be confirmed using the MINI [44], MADRS [45], ATHF [46,47], and concomitant medication record (CONMED). Trained and delegated research staff will be responsible for the informed consent process, review for eligibility, and administration of assessments.

#### Baseline.

The in-person baseline visit will begin by collecting an original ink signature on the consent form. The process of completing self-report assessments on REDCap will be explained and the visit will continue with the completion of the study’s baseline measures, such as the HDRS-17 [52], Self-Efficacy for Exercise Scale (SEE) [62], select subscales of the Physical Self Description Questionnaire – Short Form (PSDQ-S) [63], Pittsburgh Sleep Quality Index (PSQI) [64], and fitness testing (average dynamometer-measured grip strength while squeezing with maximum force for five seconds [65], number of times the participant stands from seating position in one minute [66], and number of push ups performed with correct form [67]) (Fig 1). The study team will assist participants in selecting the appropriate size of the wearable device using a sizing kit and in setting up their accounts on the mobile application. Data collection via the wearable device will begin immediately, approximately three weeks prior to the start of the intervention period, to establish baseline parameters. It will continue throughout the study for a total of approximately 13 weeks (i.e., three weeks of baseline, four weeks of intervention, and six weeks of follow-up). Daily assessments (i.e., PHQ-2 [57], GAD-2 [58], and minutes of PA during the prior day) will also be completed during the same 13 weeks. The baseline visit will end with the participant being randomized into the experimental or control group and receiving their respective study materials (i.e., the modified MoveU.HappyU Workbook for TRD or the Canadian 24-hour movement guidelines [41] handout).

#### Intervention and follow-up periods.

Participants in the experimental group will undergo a four-week remotely delivered (virtual), one-on-one, individualized PA program. At the beginning of each supervised PA session, program trainers will ask participants how many minutes of PA they engaged in during the previous week (excluding the supervised session). Trainers will also record the types of activities that the participant engaged in (with prompts to determine with whom, what, and where [e.g., indoors versus outdoors] they did). This information will be cross-referenced with the data collected by the wearable device during data analysis. At the end of each supervised PA session, program trainers will record the proportion of each type of exercise during the session, as well as participants’ responses to the Feelings Scale (FS) [68], Felt Arousal Scale (FAS) [69], and the rate of perceived exertion using the Borg Rating of Perceived Exertion Category-Ratio scale (Borg CR-10) [70]. The trainer will complete the CONMED and adverse event log with the participant. They will also collect the participant’s rating of their progress towards their PA goal from the previous week and satisfaction with the session. In the event of an adverse event, the participant will receive the necessary treatment and will be monitored by a study physician.

Participants in the control group will receive weekly phone calls from research staff during the intervention period (in lieu of the PA sessions) to complete the CONMED and adverse event log. Participants in this group will receive the same monitoring of adverse events as participants in the experimental group. These weekly phone calls will continue throughout the follow-up period.

At the end of the intervention period, all participants will complete the same self-report measures that were collected at baseline and virtually meet with the masked rater to complete the HDRS-17 [52] and fitness testing (Fig 1). Participants in the experimental group will be asked to independently continue with their PA during the six-week follow-up period. Participants in this group will also receive weekly phone calls to complete the CONMED and adverse event log during follow-up.

The final follow-up assessments occurring at week 13 will be completed in-person. Participants will access the self-reported questionnaires, perform fitness testing, and meet with the masked rater to complete the HDRS-17 [52] (Fig 1). Participants in the experimental group will also complete an exit survey to evaluate their experiences of the PA program. At this visit, participants will return the wearable device and receive the total compensation for the study (CAD $25 per in-person visit [CAD $50 total] plus an additional CAD $50 if they participated in the optional semi-structured narrative interview).

To promote participant retention and follow-up completion, participants will receive e-mail reminders 24 hours before their scheduled appointments. To ensure that participants are using the wearable device and completing assessments, weekly phone calls will be made to those with missing wearable or self-report data for three or more days in the past week. At the end of their participation in the study, participants who were randomized to the control group will be offered at least one session with a program trainer.

### Outcome measures and data collection

Outcomes will be assessed at different stages of the study (Fig 1). All assessments will occur over the phone, virtually (via video conference), or in-person (at the IPP, SMH-UHT Research Offices). Assessments will be administered by open or masked research staff (as applicable). Self-report questionnaires will be completed via REDCap.

The primary feasibility outcomes are rates of recruitment, withdrawal, and adherence. Adherence will be evaluated based on compliance with the intervention (i.e., an ordinal measure of the number of supervised PA sessions attended, as monitored by the program trainers and recorded in study logs), data completion for clinical measures, use of the wearable device (e.g., amount of data collected, missing data), and completion of the study (monitored by the study coordinator and documented in study logs). A full trial with this design will be deemed feasible if the upper 95% confidence limit for the withdrawal rate is ≤ 20%, and if the lower 95% confidence limits for the data completion and other adherence rates are ≥ 80%. If feasibility thresholds are not met, the reasons will be assessed to determine whether they could be addressed with modifications to the protocol. Data from the exit survey and semi-structured interview will be used to evaluate the acceptability of the PA intervention.

As exploratory outcomes, we will assess the efficacy of the PA program by estimating the standard deviations and within-person correlations between baseline and the end of the four-week intervention period and between baseline and the end of the six-week follow-up for the experimental and control groups. The following measures will be used to assess depressive and anxiety symptoms, and quality of life: HDRS-17 [52], Patient Health Questionnaire-9 (PHQ-9 [71]), Generalized Anxiety Disorder-7 (GAD-7 [72]), and a version of the WHO-5 [59] that has been modified for weekly administration. We will also assess the impact of the PA program on physiological biometrics, such as sleep, activity, and readiness score, which will be collected via the wearable device. The perceptions and experiences of PA in adults with TRD will be explored using qualitative data from the semi-structured narrative interviews.

### Data management, confidentiality, and monitoring

REDCap will be the main platform for data collection throughout this study. Upon enrollment, participants will be sent e-mail notifications prompting completion of self-report questionnaires via a direct link to the online data entry form. Study personnel will also complete assessments via REDCap. Data will be securely stored on the REDCap server and linked using a unique identifier for analyses. Access to REDCap will be granted to the study team via a secure web portal with multiple levels of authentication to ensure security of the data. The study coordinator will have project administrative privileges to allow for configuration of the study, management of data collection, and quality control.

Any assessments not completed via REDCap will be collected using case report forms which will be stored in a locked filing cabinet or saved on a secure server (i.e., for electronic forms). Case report forms will use a unique participant identifier for analysis. The investigator will maintain an identification list to link unique identifiers with the corresponding participant names. Great care will be taken to fully explain data collection and case report form completion during research staff training. Access to study data will be granted to authorized research staff only.

Data collected by the wearable device will be de-identified on the wearable platform using unique identifiers created by the research team to protect participants’ privacy. The data will be transferred to an external encrypted and password-protected hard drive for data analysis and storage. Access to this data will be restricted to the research team. However, participants using the wearable device will have access to their summary-level data available via the wearable device’s mobile application.

Participant interviews will be recorded using Zoom (Zoom Video Communications Inc.) [73]. The resulting audio files will be saved on a secure, password-protected, encrypted hard drive. Video recordings will be permanently deleted.

As the current study is a pilot trial with a small sample size, no data monitoring committee will be formed. Similarly, there will be no pre-specified independent audit.

### Analysis plan

Socio-demographic and clinical characteristics of the sample will be summarized by treatment group using descriptive statistics. The outcomes of feasibility and acceptability will be evaluated using counts and proportions for categorical data and means and standard deviations or medians and interquartile ranges, as appropriate, for continuous data. The proportion of participants compliant with the protocol or lost to follow-up will be estimated with 95% confidence intervals. There are no planned interim analyses.

We will estimate the means, standard deviations, and the pre-post correlations of the HDRS-17 [52], PHQ-9 [71], GAD-7 [72], and WHO-5 [59] (modified for weekly administration), and their changes per treatment group over the duration of the intervention and follow-up.

Passive data collected with the wearable device will be analyzed using data-driven techniques (e.g., a trend analysis of activity and sleep patterns). R version 4.3.3 [74] and Python (Python Software Foundation) [75] language will be used to conduct the statistical analysis.

Missing data from both the self-report measures and the wearable device will be handled based on the nature and amount of missingness. Specifically, limited missing data (i.e., < 20%) will be imputed using local linear regression and the neighbouring data points of the same measure (adjacent data points for interpolation). In cases where there is a substantial amount of consecutive missing data (i.e., > 80%), the study team will consider excluding those data points from analysis.

A combination of inductive and deductive reflexive thematic analysis will be used for all qualitative data with the aim of examining, pinpointing, and discovering patterns and developing researcher-driven themes within the data set [76,77]. The analysis will centre around researcher subjectivity, and utilize a natural and recursive coding process involving reflection and iterative engagement with the data [77].

## Discussion

PA has been recognized as an adjunctive treatment for MDD [8]. Although prior research has demonstrated the benefit of PA interventions (e.g., [6,33,78]), the literature specific to TRD remains scarce [9]. As such, more research is necessary to determine effective ways to engage this clinical population in PA. In this context, this pilot randomized controlled trial will deliver a remote, one-on-one, individualized PA program (MoveU.HappyU) to adults with TRD. The primary and secondary objectives of this study are to assess feasibility and acceptability. Exploratory objectives include estimating the efficacy of the PA program and evaluating its effect on physiological biometrics collected through a wearable device, and exploring the perceptions and experiences of PA in adults with TRD.

While few existing studies have previously delivered PA interventions as adjunctive treatments for TRD [10–13], the current trial is unique in that it will implement an intervention that is of low-demand and shorter duration (i.e., 4 weeks). The intervention also allows for individual flexibility (i.e., virtual delivery; trainers develop individualized programs for each participant; participants self-select the intensity of sessions) while also providing structured PA counselling to enact behaviour change. As such, this study will be the first to deliver MoveU.HappyU to individuals with TRD. By considering individual factors (e.g., PA preferences and priorities), MoveU.HappyU may promote PA engagement via enhanced motivation, which in turn can contribute to improvements in mental health [79,80]. Exercise intensity has implications for perceived enjoyment and can affect long-term compliance and maintenance [81]. Self-selected PA interventions produce more positive affective responses to PA and a higher sustained engagement [82]. Prior MDD research has shown that exercise at a preferred intensity gives participants a sense of control, increases enjoyment, and promotes adherence [54,83]. Supervision and delivery of PA interventions by qualified personnel also predicts lower dropout rates in psychiatric samples [84]. Moreover, previous research delivering a PA intervention that was grounded in self-determination theory to students with mild-to-moderate depression demonstrated high rates (> 90%) of adherence and improvement in depressive symptoms [85].

To the authors’ knowledge, the current study will also be the first of its kind to integrate a wearable device, paired with routine digital symptom monitoring and follow-up, into a PA intervention for TRD. A growing body of evidence supports the use of wearable devices and digital monitoring in mental health [86–88], although there is a relative paucity of research implementing these technologies in TRD. Moreover, receiving feedback and self-monitoring (e.g., via wearable activity trackers and data visualization platforms) can improve PA [37,89] and adoption of healthy behaviours more broadly [90]. Delivering MoveU.HappyU in conjunction with digital active and passive data capture will enable the evaluation of participants’ total weekly PA engagement and allow for assessment of the relationship between PA and mental health symptoms. Stability of changes in outcomes over time post-intervention may also be monitored.

While the primary focus of this pilot trial remains on evaluating the feasibility of randomizing adult participants with TRD to the PA program adjunct to TAU or TAU, participants in this study may experience some symptom relief following the intervention. MoveU.HappyU poses minimal risks to participants. To minimize the risk of sustaining injuries, the PAR-Q [49] will be completed during eligibility screening and the program will be tailored to individuals needs and capabilities. Participants will not be required to perform any PA that causes discomfort and will be instructed to discontinue the activity if they experience sharp pain, nausea, dizziness, or light-headedness. Guidance from program trainers will address any difficulties that arise while executing the individualized PA program.

This pilot study will contribute valuable insights to the literature on PA interventions for TRD, however, some limitations should be acknowledged. This trial will recruit a small number of participants (*N* = 30). While this sample size is appropriate for evaluating feasibility [50], the division of participants into small groups may introduce heterogeneity. The small sample size will also restrict the types of statistical analyses that can be conducted and preclude a definitive evaluation of the PA program’s efficacy. Moreover, due to the nature of the personalized intervention, participants randomized to the experimental group will perform various types of PA, with some participants potentially having access to exercise equipment that others may not. The contexts in which participants engage in independent PA may also differ. Variations in who participants engage in PA with and where the activities take place might influence participants’ experience of, and adherence to, the program, while also affecting the generalizability of findings. To address these differences, program trainers will record the proportions of exercises performed during the supervised sessions, and the types and context of activities performed during the unsupervised sessions. The wearable device will also track the types of activities that the participant engages in.

Data obtained through this study will be used to assess the feasibility of a trial implementing MoveU.HappyU for TRD and generate clinical parameter estimates for future larger studies. This line of research highlights the importance of PA programs that integrate personalized PA with PA counselling, influencing the development of interventions that are more tailored and effective. Future directions might include cohort studies to determine factors affecting adherence to the MoveU.HappyU intervention, or to PA interventions more broadly, in TRD. Future larger scale trials may also include additional measures of efficacy to capture mechanisms that might precede or mediate changes in depressive symptoms (e.g., positive affect, negative affect, self-efficacy) [91].

## Supporting information

S1 File SPIRIT checklist. (DOCX)

S2 File TIDieR checklist. (DOCX)

S3 File Study protocol. (PDF)

S4 File Study informed consent form. (PDF)
