# Supplementary material for: Improving adherence to physical activity in treatment-resistant depression: Protocol for a pilot randomized controlled trial of a remotely delivered program
Source: PLoS One. 2025 Sep 2;20(9):e0330848. doi: 10.1371/journal.pone.0330848 (PMC12404371; doi:10.1371/journal.pone.0330848)
Supplement: S3 File — (PDF) [file pone.0330848.s003.pdf]

Remotely Delivered Physical Activity Programme for Treatment-Resistant Depression: A Pilot Randomized Controlled Trial

Version: 3.0

**Study Principal Investigator**	Venkat Bhat, MD MSc FRCPC, St. Michael's Hospital and the University of Toronto
**Study Co-Investigators**	Catherine Sabiston, PhD, Faculty of Kinesiology and Physical Education at the University of Toronto
**Funders**	Internal funding

Clinical Study Protocol

PHYSICAL ACTIVITY PROGRAMME FOR TRD

Remotely Delivered Physical Activity Programme for Treatment-Resistant Depression: A Pilot Randomized Controlled Trial

**Investigational Products:**	4-week remotely delivered physical activity programme add-on to treatment as usual (TAU)
**Indication:**	Adult participants with treatment-resistant depression (TRD)
**Study Design:**	Study type: Interventional trial<br>Allocation: Randomized<br>Intervention model: Parallel assignment<br>Primary purpose: Feasibility and acceptability
**Principal Investigator:**	Venkat Bhat, MD MSc FRCPC DABPN<br><br>St. Michael's Hospital and the University of Toronto<br>[redacted]
**Co-Investigators:**	Catherine Sabiston, PhD<br>University of Toronto, Faculty of Kinesiology & Physical Education<br>[redacted]
**REB Study Number:**	23-069
**ClinicalTrials.Gov #**	NCT06404320
**Clinical Trial Phase:**	
**Planned Clinical Start:**	

**Planned Clinical End:**	
**Date of Protocol:**	30-Jan-2025
**Version:**	3.0

STUDY OUTLINE

Name of Sponsor-Investigator: Dr. Venkat Bhat, St. Michael's Hospital and the University of Toronto	
Name of Investigational Products: 4-week remotely delivered physical activity programme add-on to treatment as usual	Protocol Identification Code: 23-069

**Title**	Remotely Delivered Physical Activity Programme for Treatment-Resistant Depression: A Pilot Randomized Controlled Trial
**Sources of monetary or material support**	Internal Funding
**Brief title**	Physical Activity Programme for TRD
**Indication:**	Adult participants experiencing treatment-resistant depression (TRD)
**Condition(s) or focus of study**	Treatment-resistant depression (TRD)
**Number of participants**	30
**Primary outcome**	To assess the feasibility (recruitment rate, withdrawal rate, adherence rate, and data completion rate) of randomizing participants with TRD to a 4-week remotely delivered physical activity (PA) programme in addition to treatment as usual (TAU) or TAU. To assess acceptability (responses to intervention based on qualitative data from the exit survey and interview) of the PA programme.

Secondary outcomes

1. To monitor the efficacy of the implementation of a 4-week remotely delivered PA programme by assessing the improvement in depressive symptoms in participants with TRD.
2. To evaluate the effect of the PA programme on digital, physiological passive data collected via the use of a wearable device (Oura Ring[1]).
3. To explore the perceptions and experiences of PA in individuals with TRD across the lifespan.

Study design

Study type: Interventional trial
Allocation: Randomized
Intervention model: Parallel assignment
Primary purpose: Feasibility and acceptability
Phase: N/A

Masking

Outcome assessors will be blinded. Program trainer and participants will not be blinded.

Date of enrollment

Start date: Upon REB Approval
End date: Dependent on REB Approval

Eligibility criteria

Inclusion Criteria:

1. Sedentary adults (engage in less than 60 minutes of moderate-to-vigorous PA per week) between the ages of 18 and 65 years, inclusive, capable of giving informed consent.
2. Participants meeting diagnostic criteria for major depressive disorder (MDD) without psychotic symptoms according to the Diagnostic and Statistical Manual of Mental Disorders - 5th Ed. (DSM-5)[2] and currently experiencing a major depressive episode (MDE) as confirmed by the Mini International Neuropsychiatric Interview (MINI).
3. A Montgomery-Åsberg Depression Rating Scale (MADRS)[3,4] total score of ≥ 7 at screening (mild-to-severe MDE).
4. Failure of at least two trials of antidepressant therapy of adequate dose and duration during the current

episode as established by the Antidepressant Treatment History Form (ATHF)[5] and self-report.

5. Receiving treatments congruent with Canadian Network for Mood and Anxiety Treatments (CANMAT) guidelines with no changes to treatments one month before screening (28 days).[6]

Exclusion Criteria:

1. Current symptoms of mania, hypomania, mixed episodes, or psychosis.
2. Have received a diagnosis of alcohol or a substance use disorder within the past 3 months or as confirmed by the MINI.[4] Other secondary psychiatric comorbidities (e.g., anxiety disorders, trauma-related disorders, etc.) will not be excluded.
3. Pregnant females.
4. Acute risk for a cardiovascular event (i.e., cardiovascular event within the past 12 months).
5. Have any medical contraindications to exercise according to the Physical Activity Readiness Questionnaire (PAR-Q).[7]
6. Self-reported balance, gait, or locomotion difficulties that would preclude participation in a PA programme.
7. Have any other condition that, in the opinion of the investigator(s), would adversely affect the subject's ability to complete the study or its measures.
8. Have exercise-induced asthma.
9. Taking medication that interferes with heart rate response to exercise, such as beta blockers.
10. Do not own a smartphone.
11. Do not have reliable access to the Internet.
12. Have previously received intravenous ketamine treatment in the last 2 months.

Non-English-speaking individuals are excluded because the ability to communicate study information, answer questions accurately and completely about the study, and obtain consent are necessary.

**Test products, dose, and mode of administration:**	One-on-one individualized, remotely delivered PA programme for 4 weeks.
**Follow-up:**	One time in-person follow-up assessment visit 6 weeks following the interventional period with daily and weekly questionnaires using REDCap.

ABBREVIATIONS

AE	Adverse event
ATHF	Antidepressant Treatment History Form
BAS-2	Body Appreciation Scale-2
BORG-CR-10	Borg Rating of Perceived Exertion Category-Ratio Scale
CANMAT	Canadian Network for Mood and Anxiety Treatments
CONMED	Concomitant Medication Record
CRF	Case report form
DSM-5	Diagnostic and Statistical Manual of Mental Disorders - 5th Edition
GAD-2	Generalized Anxiety Disorder - 2-item scale
GAD-7	Generalized Anxiety Disorder - 7-item scale
GCP	Good Clinical Practice
HAM-D-17	Hamilton Depression Rating Scale - 17-item scale
ICF	Informed consent form
ICH-GCP	International Council for Harmonization of Technical Requirements for Pharmaceuticals for Human Use - Good Clinical Practice
IMP	Investigational medical product

IPAQ-L	International Physical Activity Questionnaire - Long Form
IPP	Interventional Psychiatry Program
MADRS	Montgomery-Åsberg Depression Rating Scale
MDD	Major depressive disorder
MDE	Major depressive episode
MINI	Mini International Neuropsychiatric Interview
PA	Physical activity
PAR-Q	Physical Activity Readiness Questionnaire
PHQ-2	Patient Health Questionnaire - 2-item Scale
PHQ-9	Patient Health Questionnaire - 9-item Scale
PII	Personal identifiable information
PSDQ-S	Physical Self Description Questionnaire – Short Form
PSQI	Pittsburgh Sleep Quality Index
RCT	Randomized controlled trial
REDCap	Research Electronic Data Capture
REB	Research Ethics Board

RPE	Rating of Perceived Exertion
SAE	Serious adverse events
SEE	Self-Efficacy for Exercise Scale
SMH-UHT	St. Michael's Hospital, Unity Health Toronto
TRD	Treatment-resistant depression
UHT	Unity Health Toronto
WHO-5	World Health Organization Well-Being Index - 5-item scale

Revision History

Version	Date	Amendment Text	Description
3.0	30-Jan-2025	Receiving treatments congruent with Canadian Network for Mood and Anxiety Treatments (CANMAT) guidelines with no changes to treatments one month before screening (28 days).[6]<br><br>The study team will also try to keep treatments unchanged during the 4-week randomized intervention phase and the 6-week follow-up phase (Sections 2.4.4 and 2.4.5).<br><br>While the study aims to avoid any changes to participants' treatment during the 4-week randomized intervention phase and the 6-week follow-up phase, changes that are required clinically to manage an acute clinical deterioration or adverse effects will be allowed and recorded; according to the treat (ITT) principle, participants whose treatment is changed will remain in the trial.<br><br>Before the intervention, research staff will confirm that participants have not recently initiated treatment with psychotherapy, brain stimulation, or ketamine. Following the ITT principle, if any changes to treatment are required, the participant will remain in the trial.	Text revised to permit treatment changes during enrollment in the study following the intent to treat principle
3.0	30-Jan-2025	An Instagram post will be made on the IPP's Instagram page. Online recruitment (e.g., social media ads) will also be facilitated through Leapcure, Inc.'s Honeybee platform. The hospital's Privacy office has approved	Added recruitment avenues

		Honeybee as a third party for recruitment provided that Honeybee is not performing pre-screening (note: as of August, 2024, Honeybee has been acquired by Leapcure, Inc., however, Honeybee's process remains the same as it was prior to the acquirement). All individuals who are interested in the study will access a pre-screening survey through REDCap which will be a preliminary measure of their eligibility for the study.	
3.0	30-Jan-2025	Research grade qualitative analysis tools (NVivo 12 or Dedoose) will be used to organize and manage the data from the verbatim transcripts	Text revised to clarify which qualitative analysis tools may be used
2.0	29-April-2024	**Aim 4:** To explore the perceptions and experiences of PA in individuals with TRD across the lifespan.	Aims of the study revised (Aim 4 added)
2.0	29-April-2024	Instead, these participants will be given a handout with the Canadian 24-hour movement guidelines and be told that they are encouraged to engage in PA.	Canadian 24-hour movement guideline handout added for control group
2.0	29-April-2024	The audio of the interview will be recorded using Zoom	Revised to record audio of interviews with Zoom
2.0	29-April-2024	During this visit, eligible participants will be asked if they would like to participate in an optional semi-structured interview as part of a narrative study (see Section 2.12.1.7). Participants who agree to participate in this optional component will be contacted by a member of the study team to schedule this interview at their convenience.<br><br>Participants will also have the opportunity to participate in an optional semi-structured interview as part of a narrative study. The target sample size will be 7-10 participants.	Added details relating to the new Aim 4; the study timeline has also been updated

		The interview will focus on collecting a story from participants about their relationship with PA and how they believe this relationship has intertwined with their depression. We will also ask participants to complete a timeline mapping exercise to recount the trajectory of their experiences with depression and PA. Time mapping is used to explore life events and trajectories across life stages and provides detailed information about transitions across one's life span. The interviews will take place at the participants convince any time during the study. Interviews are estimated to take between 60-90 minutes to complete. The audio of the interview will be recorded using Zoom and transcribed verbatim. Transcripts will be reviewed and compared to the Zoom recording to ensure verbatim transcription. Once completed, the transcripts will be sent to participants to review. Participants will be given 10 days to make any necessary edits and return the revised document to the researcher. The data analysis process will begin once the document has been reviewed and approved by the participant.<br><br>Participants who agree to participate in the optional semi-structured interview for the narrative study will additionally be compensated with $50 for their time. They will be given the total study compensation at the exit visit when they return the Oura Ring. If participants withdraw from the study, they will receive the compensation that they are entitled to when they return the Oura Ring to the study team.<br><br>Reflexive thematic analysis will be used for all qualitative data. The aim of reflexive	

		thematic analysis is […] and how TRD impacts PA engagement across one's life.	
2.0	29-April-2024	The IPP will maintain Oura Ring data ownership for all participants. This allows the IPP to delete completed study data from Oura Cloud.	Text added to clarify ownership of Oura Ring data
2.0	29-April-2024	Once a participant has completed the REDCap pre-screening form, participants who appear eligible will be sent the informed consent form (ICF), demographics form, medical history, smoking history, and family history (MSF) form, and Physical Activity Readiness Questionnaire (PAR-Q) via REDCap.<br><br>The Zoom screening visit will last around 1.5 hours. Participants will review the ICF with a member of the study team to give them additional background information on the study and associated participant responsibilities.<br><br>If the participant is deemed eligible for the study, the screening visit will end with scheduling the in-person baseline visit.<br><br>The in-person baseline visit will last around 2 hours. The visit will begin by collecting an original ink signature on the ICF and will continue with the completion of the study's baseline outcome measures.	Revised to split screening and baseline into separate visits; the study timeline and Figure 1 have also been updated; the text has additional minor modifications to reflect this change ("screening" → "baseline")
2.0	29-April-2024	Program trainers are certified PA trainers with a background in exercise and health psychology, additional training in suicide risk, and graduate-level coursework in exercise psychology and motivational interviewing.	Text revised for accuracy
2.0	29-April-2024	Participants will be recruited from the IPP and contacted via phone for a pre-screening call to give them background information on the study and associated participant	Revised placement for chronological order; added that participant's email

		responsibilities. The participant's email address will also be collected during this call if they are interested.	will be collected (already in script)
2.0	08-July-2024	Participants must have a physician (family doctor or psychiatrist) during their enrollment and participation in the trial. The investigator will inform the participant's most responsible physician about their participation in the trial.	Text added to ensure care of the participant beyond/outside of the study
2.0	08-July-2024	Removal of "Participants will no longer be eligible to continue the study if they miss more than one supervised PA session with their program trainer."	Text removed to reduce participant drop out
2.0	08-July-2024	Removal of diabetes as an exclusion criterion	Unnecessary exclusion criterion
2.0	28-Aug-2024	Receiving treatments congruent with Canadian Network for Mood and Anxiety Treatments (CANMAT) guidelines with no changes to treatments one month before screening (28 days), during the randomized intervention phase (28 days), and during the follow-up phase (42 days).	Inclusion criteria revised to encompass changes to any treatment
2.0	28-Aug-2024	At the end of their participation in the study, participants who were randomized to the control group will have access to at least one session with a PA trainer.	Revised to enhance recruitment and offer participants in the control group the opportunity to also receive personalized PA training

Table of Contents

STUDY OUTLINE 4

ABBREVIATIONS 8

1. INTRODUCTION 19

1.1 Background and Rationale 19

1.2 Objectives 20

2. METHODS 21

2.1 Trial Design 21

2.2 Eligibility Criteria 23

2.2.1 Inclusion criteria 23

2.2.2 Exclusion criteria 23

2.3 Withdrawal Criteria 24

2.4 Interventions 24

2.4.1 PA Intervention 24

2.4.2 Wearable Device (Oura Ring[1]) 26

2.4.3 REDCap 28

2.4.4 Concomitant medication 29

2.4.5 Concomitant care 29

2.4.6 Compliance 29

2.5 Outcomes 30

2.5.1 Primary outcome measures 30

2.5.2 Exploratory outcome measures 30

2.6 Participant Timeline 32

2.6.1 Study Timeline 32

2.7 Sample Size 37

2.8 Recruitment and Timeline 37

2.9 Quality Assurance 37

2.10 Allocation 37

2.11 Blinding 38

2.12 Data Collection 38

2.12.1 Trial Procedures and Evaluations 38

2.12.1.1 Pre-Study Screening 38

2.12.1.2 Screening 38

2.12.1.3 Written Consent and Baseline (in-person) 39
2.12.1.4 PA Intervention 40
2.12.5 Throughout the Study (Intervention and Follow-Up Periods) 41
2.12.1.6 Week-6 Follow-Up Assessment (in-person) 42
2.12.1.7 Qualitative Interviews 43
2.12.2 Retention 43
2.13 Data Analysis 44
2.13.1 Oura Ring Activity Analysis Plan 44
2.13.2 Qualitative Data Analysis 44
2.14 Data Monitoring 45
2.14.1 Formal committee 45
2.14.2 Interim analysis 45
2.15 Privacy, Security, and Data Confidentiality 45
2.15.1 Source documents 46
2.16 Protocol Deviations 46
2.17 Responsibilities 47
2.18 Safety/Harms 47
2.18.1 Safety of PA 47
2.18.2 AE definitions 47
2.18.3 Collection of AEs 47
2.18.4 Severity of AEs 48
2.18.5 Causality of AEs 48
2.18.6 Outcome of AEs 49
2.18.7 Procedures to manage AEs 49
2.18.8 Serious Adverse Events (SAEs) 49
2.18.9 SAE Reporting Timelines 50
2.19 Auditing 50
3. ETHICS AND DISSEMINATION 50
3.1 Ethical Standard 50
3.2 Research Ethics Board (REB) Approval 50
3.3 Protocol Amendments 51
3.4 Informed Consent Process 51
3.5 Exclusion of Minorities and Children (Special Populations) 51

3.6 Confidentiality 51
3.7 Study Discontinuation 51
3.8 Declaration of Interests 52
3.9 Ancillary and Post-Trial Care 52
3.10 Dissemination Policy 52
3.10.1 Trial Results 52
3.10.2 Authorship 52
4. STUDY ADMINISTRATION 52
4.1 Key Contacts 52
4.2 Funding 52
4.3 Roles and Responsibilities 53
4.3.1 Sponsor and Funding 53
5. REFERENCES 53

1. INTRODUCTION

1.1 Background and Rationale

Over 300 million people worldwide experience major depressive disorder (MDD).[8] More than one-third of these patients will not respond to at least two antidepressant medication trials, meeting the criteria for treatment-resistant depression (TRD).[9] As a result, alternative therapeutic modalities, such as exercise, are garnering interest.[10] Numerous studies have reported an association between physical activity (PA) and improvements in mood and mental health[11–13] in both clinical and non-clinical populations.[14]

Prior research demonstrates that the impact of PA depends on the frequency,[15,16] intensity,[17,18] and domain.[19] The optimal dose of PA (i.e., frequency, intensity, duration, and domain) has yielded mixed results. Some studies have found light-intensity to be just as effective as moderate-intensity PA.[20] However, others have found an association between low-intensity PA and lower prevalence of depressive symptoms, but not moderate- or vigorous-intensity PA.[21] Low doses and enjoyability associated with PA may play a more significant role in the protective effect of PA on mental health and well-being.[22] In line with this, a recent review suggested that self-selected intensity may have a greater protective effect on depression symptoms.[23]

The mechanism underlying the antidepressant effect of exercise is multifaceted. Physiologically, exercise activates the endocannabinoid system,[24] and modulates endorphin,[25] neurotrophin, and monoamine levels[26] to improve cognitive deficits and affective symptoms of depression. Exercise induces the release of brain-derived neurotrophic factor, which promotes brain neurogenesis and contributes to a reduction in depressive symptoms and improvement in cognitive functioning.[26] In doing so, PA diverts an individual's attention away from negative preoccupations[27] and improves perceptions of self-efficacy[28] and self-concept[29] to ultimately contribute to an improvement in treatment outcomes as well. To be more specific, regular PA participation can give individuals a sense of mastery and allow them to experience new bodily sensations such as an improved body image, sense of achievement, and feelings of control that may successfully distract them from their negative thoughts and reduce depressive symptoms.[30]

Despite the wide-reaching benefits of PA for mental health, few studies to date have examined the role of PA in reducing depression symptoms in adult TRD patients.[31,32] These studies demonstrate the need for supervision and personalization in exercise interventions for patients to optimize mental health outcomes for each individual.[33,34] By acknowledging and incorporating individual PA preferences and priorities, we can increase PA frequency via enhanced motivation and engagement and thus contribute to improvements in mental health.[22,35] In addition, a recent systematic review identified that the quality of research reporting and conduct across randomized controlled trials (RCT) relating to exercise interventions was suboptimal compared to that of pharmacological RCTs,[36] thus demonstrating a need for higher quality research. As a result, in collaboration with the University of Toronto, we will conduct a feasibility and acceptability pre-

post study examining the delivery of a remote one-on-one individualized PA programme in adult participants with TRD. Participants who demonstrate interest or are referred from the Interventional Psychiatry Program (IPP) will be offered supervised PA training once a week and unsupervised PA training 2-3 times/week for 4 weeks, plus PA counselling, to enhance motivation to engage in PA without supervision. We will evaluate the PA intervention using a comprehensive public health framework, RE-AIM.[37] This study will target various program indicators including, reach, efficacy, adoption, implementation and maintenance of the PA intervention. In addition to the exit survey, participants will complete a semi-structured qualitative interview with a research team member to better understand their experience of participating in the PA intervention.

1.2 Objectives

Over a 13-week (~3-week baseline period, 4-week randomized treatment phase, and 6-week follow-up) period, we will conduct a pilot randomized controlled clinical trial to determine the feasibility of a trial randomizing adult participants with TRD to a remotely delivered one-on-one individualized PA programme in addition to treatment as usual (TAU) or TAU. We will also assess the acceptability of the PA programme. We aim to include the use of a digital platform (i.e., the Oura Ring[1]) to evaluate the effect of the PA programme on digital passive data collected. This study has the following aims:

Primary Aims:
Aim 1: To assess the feasibility (recruitment rate, withdrawal rate, adherence rate, and data completion rate) of randomizing adult participants with TRD to a PA programme add-on to TAU or TAU and to assess acceptability (responses on the exit survey and semi-structured qualitative interviews) of the PA programme.

> ***H1a (Recruitment rate):*** Over 15 months, we will be able to recruit and assess 2 participants per month.
> ***H1b (Withdrawal rate):*** The overall withdrawal rates will be no more than 20%.
> ***H1c (Data completion rate):*** The data completion rates will be more than 80% in both treatment arms.
> ***H1d (Adherence rate):*** Adherence rates, including treatment compliance and study completion, will be more than 80% in both treatment arms.
> ***H1e (Acceptability):*** Participants will report satisfaction with the content and delivery of the intervention, as measured by the exit survey and semi-structured qualitative interview.

If feasibility thresholds are not met, our team will evaluate the reasons and determine whether they could be addressed with protocol modifications.

Exploratory Aims:

Aim 2: To monitor the efficacy of the implementation of a 4-week remotely delivered PA programme by assessing the change in depressive symptoms, anxiety symptoms, and quality of life in participants with TRD and calculating the difference between scores at these time points for each group.

Q2a: What are the estimates for the standard deviation of Hamilton Depression Rating Scale – 17 item (HAM-D-17)[38] and its within-person correlation between baseline and the end of the 4-week intervention period and between baseline and the end of the 6-week follow up?

Q2b: What are the estimates for the standard deviation of Patient Health Questionnaire – 9 item (PHQ-9)[39] and its within-person correlation between baseline and the end of the 4-week intervention period and between baseline and the end of the 6-week follow up?

Q2c: What are the estimates for the standard deviation of Generalized Anxiety Disorder – 7 item (GAD-7)[40] and its within-person correlation between baseline and the end of the 4-week intervention period and between baseline and the end of the 6-week follow up?

Q2d: What are the estimates for the standard deviation of World Health Organization Well-Being Index – 5-item (WHO-5)[41] and its within-person correlation between baseline and the end of the 4-week intervention period and between baseline and the end of the 6-week follow up?

Aim 3: To evaluate the effect of the PA programme on digital physiological passive data collected through the use of a wearable device (Oura Ring[1]).

Aim 4: To explore the perceptions and experiences of PA in individuals with TRD across the lifespan.

2. METHODS

2.1 Trial Design

We propose an interventional, randomized, 4-week pilot clinical trial. The trial will enroll 30 participants to generate data for feasibility and acceptability, and to estimate variability of the efficacy outcomes to use in the sample size calculation for future larger scale trials. We will evaluate the feasibility and acceptability of a 4-week remote one-on-one individualized PA programme for participants with TRD (i.e., ≥ 2 failed trials of antidepressant treatment) and in a mild-to-severe major depressive episode (MDE) as measured by a Montgomery-Åsberg Depression Rating Scale (MADRS) total score of ≥ 7, while simultaneously using a digital wearable device (Oura Ring) to monitor participant's physiological data.

Participants will be randomized to one of two treatment arms:

1) PA group: Participants in this group will continue TAU and will participate in a 4-week remotely delivered one-on-one individualized PA programme.

OR

2) TAU group: Participants in this group will continue to receive TAU, but will not receive the PA programme add-on. Instead, these participants will be given a handout with the Canadian 24-hour movement guidelines[42] and be told that they are encouraged to engage in PA.

Participants in both groups will use a wearable device, known as an Oura Ring[1], to collect passive data related to physiological variables (e.g., sleep, heart rate, activity). Please see Section 2.4.2 for more information on the Oura Ring and Section 2.4.1 for a thorough description of the remotely delivered PA programme. **Figure 1** summarizes the proposed study design. Participants in both treatment arms will continue to receive treatment congruent with Canadian Network for Mood and Anxiety Treatments (CANMAT) guidelines[6] with no changes to treatments 28 days before screening. The study team will also try to keep treatments unchanged during the 4-week randomized intervention phase and the 6-week follow-up phase (Sections 2.4.4 and 2.4.5).

At the end of their participation in the study, participants who were randomized to the TAU group will have access to at least one session with a PA trainer.

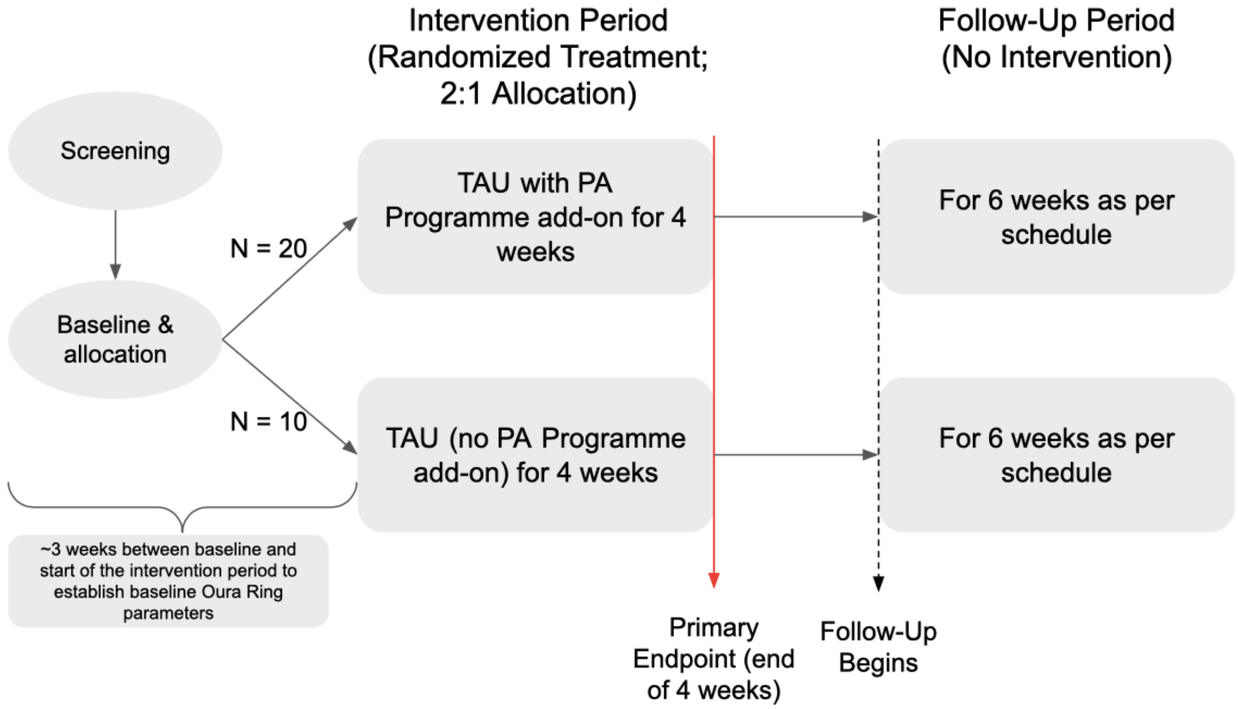

Figure 1. Proposed study design. TAU = treatment as usual, PA = physical activity.

2.2 Eligibility Criteria

2.2.1 Inclusion criteria

1. Sedentary adults (engage in less than 60 minutes of moderate-to-vigorous PA per week) between the ages of 18 and 65 years, inclusive, capable of giving informed consent.
2. Participants meeting diagnostic criteria for MDD without psychotic symptoms according to the Diagnostic and Statistical Manual of Mental Disorders - 5th Ed. (DSM-5)[2] and currently experiencing a MDE as confirmed by the Mini International Neuropsychiatric Interview (MINI).[4]
3. A MADRS[3] total score of ≥ 7 at screening (mild-to-severe MDE).
4. Failure of at least two trials of antidepressant therapy of adequate dose and duration during the current episode as established by the Antidepressant Treatment History Form (ATHF)[5] and self-report.
5. Receiving treatments congruent with CANMAT[6] guidelines with no changes to treatments one month before screening (28 days).

2.2.2 Exclusion criteria

1. Current symptoms of mania, hypomania, mixed episodes, or psychosis.
2. Have received a diagnosis of alcohol or a substance use disorder within the past 3 months or as confirmed by the MINI.[4] Other secondary psychiatric comorbidities (e.g., anxiety disorders, trauma-related disorders, etc.) will not be excluded.
3. Pregnant females.
4. Acute risk for a cardiovascular event (i.e., cardiovascular event in the past within the past 12 months).
5. Have any medical contraindications to exercise according to the Physical Activity Readiness Questionnaire (PAR-Q).[7]
6. Self-reported balance, gait, or locomotion difficulties that would preclude participation in a PA programme.
7. Have any other condition that, in the opinion of the investigator(s), would adversely affect the subject's ability to complete the study or its measures.
8. Have exercise-induced asthma.
9. Taking medication that interferes with heart rate response to exercise, such as beta blockers.
10. Do not own a smartphone.
11. Do not have reliable access to the Internet.
12. Have previously received intravenous ketamine treatment in the last 2 months.

Non-English-speaking individuals are excluded because the ability to communicate study information, answer questions accurately and completely about the study, and obtain consent are necessary.

2.3 Withdrawal Criteria

Participants are free to withdraw their consent and/or end their participation in the research study at any time without penalty or loss of benefits or treatment to which they are otherwise entitled. Participants may be withdrawn from the study by the participant's doctor if they feel that it is in their best interest.

Participation may also be discontinued if:

- The participant fails to adhere to the study's responsibilities
- The participant meets any exclusion criteria (either newly developed or not previously recognized)
- The participant presents with any severe adverse event (AE)
- Anything, in the opinion of the investigator(s), that would place the participant at increased risk or preclude the participant's full compliance with or completion of the study

If a participant experiences an AE, they will receive the necessary treatments to alleviate any untoward event and will be monitored by the study physician until it is safe for them to be discharged. Participants will be followed up with the next day to monitor the progression of their condition.

Data from a participant who has been withdrawn will be collected up to the next day after the withdrawal to ensure that the participant has no more complications. All data collected before the day of withdrawal will be used for analysis. If a participant withdraws from the study at any time, the reasons for withdrawal will be collected and documented as part of the feasibility outcomes for this study.

2.4 Interventions

2.4.1 PA Intervention

The design of the PA intervention[43] involves a pragmatic methodological approach including non-restrictive eligibility for participation, leaving details of the intervention implementation up to the program trainer (individualized PA program) and allowing the intervention sessions to be conducted by individuals from a variety of backgrounds and levels of experience. Participants in the PA group will undergo a 4-week remotely delivered one-on-one individualized PA programme that will involve weekly 1 hour-long sessions with their program trainer. Participants will be matched with a program trainer and work individually with the trainer for the duration of 4 weeks. Program trainers are certified PA trainers with a background in exercise and health psychology, additional training in suicide risk, and graduate-level coursework in exercise psychology and motivational interviewing.

Prior to the beginning of the intervention, participants will participate in a goal-setting session and will be given a personalized introduction to the program. During this session, participants will meet their program trainer virtually and they will discuss their goals, needs, equipment access, and previous PA experience with the program trainer. After this meeting, the program trainer will develop an individualized and tailored PA program for the participant.

During the intervention period, participants will meet with the program trainer once a week virtually over Zoom for 4 weeks. During these weekly Zoom sessions, participants will engage in 30 minutes of behavioral change coaching with the program trainer using a version of the MoveU.HappyU Workbook that has been adapted for TRD. This behavioral change coaching will involve participants setting weekly goals, assessing goal progression, developing action plans, exploring barriers and enablers to goals, and setting cues or prompts for self-directed PA. After the first 30 minutes, participants will engage in 30 minutes of a structured PA program that is tailored to each participant's needs and goals. The intensity of the PA sessions will be self-selected.

Prior to the start of each PA session, program trainers will ask participants how many minutes of PA they engaged in during the week (excluding the supervised session) as well as the type of activities they engaged in (prompts to include *with who, what, and where [e.g., indoors vs. outdoors]*). This information will be recorded in Research Electronic Data Capture (REDCap) and cross-referenced with the data collected by the Oura App.

Although the PA programme will differ from participant to participant, the overall organization of the weekly PA sessions will be standardized across participants. Specifically, the PA sessions will involve a warmup consisting of 5-7 minutes of light to moderate intensity cardiorespiratory and endurance activities, a conditioning component consisting of 20 minutes of individualized aerobic or resistance exercises and a 3-5 minute cool down/stretch. PA sessions will target major muscle groups such as the chest, shoulders, upper and lower back, abdomen, hips and legs) with a specific emphasis on proper form and technique (e.g., deliberate movements in a controlled manner, moving through the full range of motion of the joint, and utilizing proper breathing techniques). Since the PA program will involve a combination of aerobic and resistance exercises, program trainers will record the proportion/percentage of each type of exercise during the session on REDCap. The Rate of Perceived Exertion (RPE) using the Borg Rating of Perceived Exertion Category-Ratio Scale (BORG-CR-10)[44] scale will be recorded at the end of each supervised session by the program trainer to measure perceived effort and affective valence (i.e., pleasure/displeasure of exercise). Additionally, the Talk Test[45] will be used to encourage engagement in moderate-to-vigorous intensity PA. In addition, participants will complete the Feelings Scale[46] and the Felt Arousal Scale[47] and responses will be recorded by the program trainer on REDCap.

After each session, participants are also asked to complete homework and rate how satisfied they were with the delivery of the session using the adapted MoveU.HappyU Workbook. Their satisfaction rating will be recorded on REDCap by the program trainer. Participants will be

instructed to engage in PA for 150 minutes in total, at 50-65% of their maximum work capacity, each week. This can be done by practicing PA between 3-5 times per week. It is important to note that participants are only engaging in PA with the program trainer once per week for 30 minutes. The remaining 120 minutes of PA will be completed independently by the participant, on their own time, according to the tailored PA program developed by their program trainer. Upon completion of the program, participants will complete an exit survey to evaluate their experiences of the program. Additionally, they will meet with a member of the study staff for a semi-structured interview to further discuss the acceptability of the PA program.

2.4.2 Wearable Device (Oura Ring[1])

In this study, we will use a digital wearable device to continuously collect longitudinal, passive data that will help us monitor activity, sleep, and physiological data related to stress and mental health symptoms.[48] For this, we will use the commercially available wearable device, Oura Ring,[1] which collects sleep and activity data, as well as a series of physiological signals that may be potentially relevant in the context of PA and mental health. This device is identified as a passive wearable device that can be used without obstructing one's daily life. Detailed information on the sensors and vital signs collected by Oura Ring are summarized in Table 1.

Table 1. Description of the wearable device to be used and vital signs to be collected

Description	Data Transfer	Data elements
The Oura Ring[1] is a smart ring that is worn on the finger and collects heart rate and respiration rate from an individual. It uses advanced sensor technology to allow for precise, personalized health insights about one's body. This device measures daily activity, oxygen saturation, heart rate, heart rate variability, and body temperature variation. Additionally, the device	Participants will download and log on to the Oura Ring[1] mobile app using the study email credentials. The Oura Ring[1] connects to a user's smartphone via Bluetooth. It will collect data locally and send it to the mobile app when synced. Once the Oura Ring[1] app has internet access, its data will be transferred to and stored on its respective servers.	● Heart rate<br>● Respiratory rate<br>● Daily activity<br>● Heart rate variability<br>● Oxygen saturation<br>● Body temperature variation (delta)<br>● Individual's readiness<br>● Sleep<br>● Activity

scores an individual's readiness, sleep, and activity.		

Upon acceptance to the study, the Oura Ring[1] wearable device will be made available with its respective Privacy Policy for participants to read. Passive data collection on the wearable device will begin approximately 3 weeks before the interventional period begins (to establish baseline parameters). The procedure for the use of the wearable device is as follows:

(1) Participants will be offered to consent to collecting data from the Oura Ring[1] wearable device.
(2) Members of the research team will create secure and unique IDs, dummy emails, and passwords to be given to each participant for de-identification.
(3) Participants will collect the Oura Ring[1] wearable device (during the baseline visit), download its respective mobile app on their personal device, and log in using the given email and password.
(4) The duration of participants using the digital platform will comprise approximately 13 weeks.
(5) Once participants have completed the study, the devices and accounts will be returned, cleaned, and formatted. Participant data will only be accessible by the research team and the participant using it.
    (a) Participants will return the ring and charger to the IPP at St. Michael's Hospital, Unity Health Toronto (SMH-UHT) during their final assessment visit.

The participant will pick up the Oura Ring wearable device at the IPP at SMH-UHT during their scheduled baseline visit. At that time, the study team will assist the participant with selecting the appropriate ring size (using a sizing kit) and setting up their account on the Oura mobile application. Participants will have access to the study coordinator's contact information in case any technical problems and/or questions arise.

The wearable device will be evaluated for its feasibility to collect physiological data in naturalistic settings (i.e., dropout rates, amount of data collected, missing data). It will be helpful in tracking individual trends over time, which will potentially provide us with a better understanding of physiological responses related to PA and depression. These multivariate models will help us build prediction tools, which could be an important factor in the design of decision support tools related

to mental health. We will incorporate sex/gender- and age-based analysis throughout the study to adjust the models as needed.

The description of the physiological signals to be collected by the Oura Ring[1] is provided below:

1. Sleep information: The Oura Ring[1] performs sleep analysis and stores a set of measurement parameters that summarize each period. The ring calculates the sleep period specific parameters within four hours from the period end, but sleep analysis is always triggered when participants open the application. These parameters include sleep related data such as bedtime, total duration, awake, light, rapid eye movement, and deep sleep durations. It also collects physiological signals such as heart rate, respiratory rate, heart rate variability, and body temperature changes.
2. Activity information: Activity summary contains daily activity summary values and detailed activity levels. Activity levels are expressed in metabolic equivalent of task minutes (metabolic equivalent minutes). The Oura Ring[1] tracks activity based on movement and presents the activity score based on the duration of low, medium, and high activity levels. The movement is also used for a daily step count and an estimation of calories.
3. Readiness information: Readiness score is interpreted from sleep, activity, resting heart rate, heart rate variability, recovery index, and body temperature scores. A readiness score above 85% indicates that the wearer is well recovered. A score below 70% usually means that an essential readiness contributor, such as body temperature or previous night’s sleep, falls outside a wearer’s normal range, or clearly differs from recommended, science-based values.

2.4.3 REDCap

REDCap (https://www.project-redcap.org) software supported by the Applied Health Research Centre at Unity Health Toronto (UHT) will be used for data collection and overall study data management over the course of this project. REDCap is an open-source, web-based clinical data management and electronic data capture system and database. The system is developed and managed in compliance with UHT privacy, the Health Insurance Portability and Accountability Act, the Personal Information Protection and Electronic Documents Act, and Food and Drug Administration 21 Code of Federal Regulations Part 11 regulations, providing functions such as defined user roles and privileges, user authentication and encryption for in-transit data, de-identification of protected health information and comprehensive auditing features to record and monitor access and changes to data. This system will be used to send scheduled questionnaires to participants and store active data and data monitoring and for the query and export of datasets for statistical analysis and modeling.

Access to REDCap will be secured through a secure web portal and protected by multiple levels of authentication. A Project Coordinator will be assigned the project administrative privileges for study configuration, data collection management, and quality control.

Upon acceptance to the study, participants will complete mental health questionnaires on a daily and weekly basis. To complete these questionnaires, participants will receive an email notification with the REDCap survey link to their personal email address. Upon receiving the link, participants will answer the self-report questionnaires provided through the link. Active data will be sent to and stored on the REDCap server and will be linked by the unique ID for final analysis. Access to this data will only be granted to the project administrator to download the data from the REDCap server for analysis.

2.4.4 Concomitant medication

Before the intervention, research staff will document all medications taken by participants on a regular basis. No changes to treatment are allowed one month (28 days) before screening due to the probable impact on psychiatric symptoms. Participation in the study will not alter the standard of care, including any required changes to treatment regimens. While the study aims to avoid any changes to participants' treatment during the 4-week randomized intervention phase and the 6-week follow-up phase, changes that are required clinically to manage an acute clinical deterioration or adverse effects will be allowed and recorded; according to the treat (ITT) principle, participants whose treatment is changed will remain in the trial.

2.4.5 Concomitant care

Before the intervention, research staff will confirm that participants have not recently initiated treatment with psychotherapy, brain stimulation, or ketamine. Following the ITT principle, if any changes to treatment are required, the participant will remain in the trial.

2.4.6 Compliance

Similar studies among clinical TRD samples have shown promising compliance rates of 91%[31] and 94%.[32] Even though the weekly PA sessions via Zoom will be supervised, the other sessions will be unsupervised. As such, treatment adherence will not be known with 100% accuracy but can be estimated using self-report validated by Oura Ring data. Nevertheless, prior studies have shown that exercise interventions in which participants are able to self-select exercise intensity promote treatment adherence.[49,50] The introduction of exercise regimes that increase in intensity incrementally and give the participants control over the level of intensity tend to increase participants' enjoyment of the exercise and thus increase compliance rates.[49] Participant compliance will be assessed on a weekly basis during the PA sessions with the program trainer using the adapted MoveU.HappyU Workbook wherein participants will be asked to rate their progress towards their PA goal from the previous week. In addition, program trainers will ask participants the number of minutes they spent engaging in PA during the week (excluding the

supervised session) and the kinds of activities they engaged in. Additionally, to ensure that participants are completing the scales and using the Oura Ring as required, weekly compliance calls will be made to participants who are missing Oura data for 3 or more days in the past week or if REDCap data is missing.

2.5 Outcomes

All outcomes will be assessed at different stages of the study. Please refer to the study's timeline, Section 2.6.1, for a detailed depiction of the nature and frequency of all assessments. All study personnel administered scales will occur via phone call, Zoom or in-person by trained blinded (as applicable) study staff members. Self-report questionnaires will be completed via REDCap.

2.5.1 Primary outcome measures

To evaluate the feasibility of randomizing adult participants with TRD to a PA programme add-on to TAU or TAU, primary outcomes will include recruitment rate (minimum threshold: 2 participants/month), withdrawal rate (maximum threshold: 20%), data completion rate (minimum threshold: 80%) and adherence rate (minimum threshold: 80%). Qualitative data from the exit survey and semi-structured interview will be used to evaluate the acceptability of the PA intervention. A full trial will be considered feasible with this design if the upper 95% confidence limit for withdrawal rate is ≤ 20% and both of the lower 95% confidence limits for data completion rate and adherence rate are ≥ 80%. If feasibility thresholds are not met, our team will evaluate the reasons and determine whether they could be addressed with protocol modifications. Data on the primary endpoints will be collected and recorded throughout the study. Tabulation and analysis will occur after all study-related data have been collected and follow-up of all study subjects is complete.

In addition to the exit survey, semi-structured qualitative interviews will be conducted with the participants to determine the acceptability of the PA intervention. Interviews will be conducted one-on-one with a designated member of the study staff. Interviews will be recorded (with the participants' permission), transcribed verbatim, and analyzed using reflexive thematic analysis[51,52] to explore the participant's experiences in the PA program. The audio of the interview will be recorded using Zoom and will be stored on an encrypted, password-protected hard drive.

2.5.2 Exploratory outcome measures

<u>Aim 2: Efficacy of the implementation of a 4-week remotely delivered PA programme.</u>

<u>Depression:</u>

a) Estimates for the standard deviation of HAM-D-17[38] and its within-person correlation between baseline and the end of the 4-week intervention period and between baseline and the end of the 6-week follow up for the PA and control group.

b) Estimates for the standard deviation of PHQ-9[39] and its within-person correlation between baseline and the end of the 4-week intervention period and between baseline and the end of the 6-week follow up for the PA and control group.

Anxiety:

a) Estimates for the standard deviation of GAD-7[40] and its within-person correlation between baseline and the end of the 4-week intervention period and between baseline and the end of the 6-week follow up for the PA and control group.

Quality of Life:

a) Estimates for the standard deviation of WHO-5[41] and its within-person correlation between baseline and the end of the 4-week intervention period and between baseline and the end of the 6-week follow up for the PA and control group.

Aim 3: Effect of the PA programme on digital physiological passive data (e.g., sleep, activity and readiness information; see Section 2.4.2) collected through the use of a wearable device.

Aim 4: To explore the perceptions and experiences of PA in individuals with TRD across the lifespan.

2.6 Participant Timeline

2.6.1 Study Timeline

	**Active Study**																
	Screening	Allocation & Baseline	Buffer	Intervention												Follow-Up	
**Week**	0	1	2-3 MUHU Session 1	4 MUHU Session 2			5 MUHU Session 3			6 MUHU Session 4			7 MUHU Session 5			8-13	
**Frequency**				With Trainer	Weekly	Daily	With Trainer	Weekly	Daily	With Trainer	Weekly	Daily	With Trainer	Weekly	Daily	Daily	Weekly
	**Eligibility Screening**																
Consent	X																
DEMO	X																
MINI	X																
ATHF	X																

MSF	X																
MADRS	X																
PAR-Q	X																
Oura Ring Pick-up		X															
	**Interventions**																
A - PA programme + TAU				X	X	X	X	X	X	X	X	X	X	X	X		
B - TAU				X	X	X	X	X	X	X	X	X	X	X	X		
	**Clinical Assessments**																
HAM-D-17		X												X		X[2]	
CONMED	X	X		X[1]			X[1]			X[1]			X[1]				X
AE				X[1]			X[1]			X[1]			X[1]				X
	**Active Data (REDCap)**																
GAD-7		X												X		X[2]	

GAD-2		X	X			X			X			X			X	X	
PHQ-2		X	X			X			X			X			X	X	
PHQ-9		X												X		X[2]	
WHO-5		X			X			X			X			X			X
FS				X			X			X			X				
FAS				X			X			X			X				
BORG-CR-10				X			X			X			X				
IPAQ-L		X			X			X			X			X			X
BAS-2		X												X		X[2]	
Body-related self-conscious emotions		X												X		X[2]	
SEE		X												X		X[2]	
PSDQ-S		X												X		X[2]	
PSQI		X												X		X[2]	

	**Functional Fitness Testing**																
Grip Strength		X														X[2]	
Sit to Stand		X												X		X[2]	
Push Up		X												X		X[2]	
	**Passive Wearable Data (Oura Ring)**																
Sleep Information		X	X			X			X			X			X	X	
Activity Information		X	X			X			X			X			X	X	
Readiness Information		X	X			X			X			X			X	X	
Physiological Data		X	X			X			X			X			X	X	
	**Qualitative Assessments**																
Exit Survey																X[2]	
Qualitative Interview	X[3]															X[4]	
	MUHU = Adapted MoveU.HappyU<br>DEMO = Demographics Form<br>MINI = Mini-international neuropsychiatric interview<br>ATHF = Antidepressant Treatment History Form<br>MSF = Medical history, smoking history, family history									GAD-7 = Generalized Anxiety Disorder - 7-item scale<br>GAD-2 = Generalized Anxiety Disorder - 2-item scale<br>PHQ-2 = Patient Health Questionnaire - 2-item scale<br>WHO-5 = World Health Organization Five Well-Being Index<br>BAS-2 = Body Appreciation Scale-2							

	HAM-D-17 = Hamilton Depression Rating Scale – 17-Item<br>TAU = Treatment as usual<br>MADRS = Montgomery-Åsberg Depression Rating Scale<br>AE = Adverse Event Log<br>CONMED = Concomitant medication record<br>PAR-Q = Physical Activity Readiness Questionnaire<br>FS = Feelings Scale<br>FAS = Felt Arousal Scale<br>BORG-CR-10 = Borg Rating of Perceived Exertion (RPE) Category-Ratio Scale<br>IPAQ-L = International Physical Activity Questionnaire - Long Form<br>PHQ-9 = Patient Health Questionnaire –9-item scale	SEE = Self-Efficacy for Exercise Scale<br>PSDQ-S = Physical Self Description Questionnaire – Short Form<br>PSQI = Pittsburgh Sleep Quality Index<br>Sleep Information = bedtime, total duration, awake, light, rapid eye movement, and deep sleep durations, heart rate, respiration rate, heart rate variability, and temperature changes).<br>Activity Information = daily activity summary values, detailed activity levels (metabolic equivalent minutes), step count, and estimated calories.<br>Readiness Information = interpreted from sleep, activity, resting heart rate, heart rate variability, recovery index, and temperature scores.<br>Physiological Data = heart rate, heart rate variability, body temperature variation, oxygen saturation, respiratory rate

Note: X^1 = the CONMED and AE log will occur during the supervised PA session for participants randomized to the PA group; participants in the TAU group will instead receive a weekly phone call. X^2 = will occur once at the Exit Visit with Oura Ring drop off. X^3 = optional qualitative interview to occur any time during study. X^4 = the qualitative interviews will occur once during the 6-week follow-up period.

2.7 Sample Size

Because the anticipated effect size is unknown, a formal sample size calculation cannot be completed. For this interventional pilot trial, it is anticipated that 30 participants will be recruited.

2.8 Recruitment and Timeline

The target recruitment rate is at least 2 participants per month for a total of 30 subjects recruited over 15 months. Participants will be recruited from the IPP and contacted via phone for a pre-screening call to give them background information on the study and associated participant responsibilities. The participant's email address will also be collected during this call if they are interested. Other outlets of recruitment will also be used such as advertisements (flyers) that will be posted in UHT affiliated hospitals. An Instagram post will be made on the IPP's Instagram page. Online recruitment (e.g., social media ads) will also be facilitated through Leapcure, Inc.'s Honeybee platform. The hospital's Privacy office has approved Honeybee as a third party for recruitment provided that Honeybee is not performing pre-screening (note: as of August, 2024, Honeybee has been acquired by Leapcure, Inc., however, Honeybee's process remains the same as it was prior to the acquirement). All individuals who are interested in the study will access a pre-screening survey through REDCap which will be a preliminary measure of their eligibility for the study.

Participants must have a physician (family doctor or psychiatrist) during their enrollment and participation in the trial. The investigator will inform the participant's most responsible physician about their participation in the trial.

2.9 Quality Assurance

Methods for quality assurance will be strictly observed. A start-up meeting involving all study site staff, co-investigators, and partners will be held prior to the launch of the study. Further training will be held prior to commencing the study. During training, great care will be taken to fully explain the study procedures and case report form (CRF) completion.

2.10 Allocation

If potential participants meet the eligibility criteria, they will be enrolled in the trial and will be randomized in a 2:1 allocation to receive a PA program add-on (experimental group) or TAU (control group). Thus, 20 participants will be randomized to the PA group and 10 participants will be randomized to the TAU group. Block randomization will be computer-generated, and the sample will be stratified for sex given the potential for differences in response in these groups. The randomization schedule will employ random permuted blocks of varying sizes. It will be administered by a member of the study staff.

2.11 Blinding

Outcome assessors and data analysts will be blinded to the group assignments. Research personnel supervising PA sessions (program trainers) and participants will be unblinded, as they will be participating in the training sessions. Research personnel contacting participants for the HAM-D-17 will be blinded. To maintain blinding, participants will be told that the members of the research team (excluding the program trainer) are unaware of whether they are assigned to the PA or TAU group and as such, will be instructed to not discuss details of which group they've been assigned to when responding to questions asked during clinical assessments. To evaluate the success of the blinding, research personnel completing the HAM-D-17 with participants will complete a form wherein they will guess which group the participant is randomized to and this will be compared with the actual group assignment.

2.12 Data Collection

The IPP at SMH-UHT will be the only site where we will further evaluate individuals eligible for the trial. The study team will be based at SMH-UHT and the University of Toronto.

2.12.1 Trial Procedures and Evaluations

2.12.1.1 Pre-Study Screening

As outlined in Section 2.8, potential participants will access a pre-screening survey through REDCap which will be a preliminary measure of their eligibility for the study. Once an individual has completed the REDCap pre-screening form, those who appear eligible will be sent the informed consent form (ICF), demographics form, medical history, smoking history, and family history (MSF) form, and Physical Activity Readiness Questionnaire (PAR-Q)[7] via REDCap. If the individual agrees to participate in the study, they will digitally sign/write their name on the REDCap ICF which will stand in place of a signature. An original ink signature will be collected at the in-person baseline visit. At the time when REDCap links to the ICF, demographics form, MSF, and PAR-Q are sent to participants, participants will also be prompted to schedule their Zoom screening visit using Calendly,[53] an automated scheduling system, should they agree to participate. Calendly[53] will be used throughout the duration of the study to schedule study visits with both study staff and program trainers. Alternatively, study visits can be scheduled via email.

2.12.1.2 Screening

The Zoom screening visit will last around 1.5 hours. Participants will review the ICF with a member of the study team to give them additional background information on the study and associated participant responsibilities. Participants' eligibility will be confirmed using the measures listed below.

1. The Montgomery-Åsberg Depression Rating Scale (MADRS).[3] In order for participants to be eligible for the study, their total score should be ≥ 7 (mild-to-severe MDE).

2. The Mini International Neuropsychiatric Interview (MINI)[4]
3. The Antidepressant Treatment History Form (ATHF)[5]
4. Concomitant Medication Record (CONMED)
5. Participants will be asked to answer questions about the consent form to determine that the study process and the duration of participation are completely understood by all participants

During this visit, eligible participants will be asked if they would like to participate in an optional semi-structured interview as part of a narrative study (see Section 2.12.1.7). Participants who agree to participate in this optional component will be contacted by a member of the study team to schedule this interview at their convenience.

If the participant is deemed eligible for the study, the screening visit will end with scheduling the in-person baseline visit.

2.12.1.3 Written Consent and Baseline (in-person)

The in-person baseline visit will last around 2 hours. The visit will begin by collecting an original ink signature on the ICF and will continue with the completion of the study's baseline outcome measures. The process of completing self-report assessments on REDCap will be explained. Participants will access the baseline self-reported questionnaires through REDCap during their in-person visit. The details of the frequency of all assessments and measures are provided in the study timeline (Section 2.6.1). During this visit participants will complete:

1. Patient Health Questionnaire - 9-item Scale (PHQ-9)[39]
2. Patient Health Questionnaire - 2 item (PHQ-2; modified for daily administration)[54]
3. Generalized Anxiety Disorder - 7-item Scale (GAD-7)[40]
4. Generalized Anxiety Disorder Scale - 2 item (GAD-2; modified for daily administration)[55]
5. World Health Organization-Five Well-Being Index (WHO-5; modified for weekly administration)[41]
6. International Physical Activity Questionnaire - Long Form (IPAQ-L)[56]
7. Body Appreciation Scale-2 (BAS-2)[57]
8. Body-related self-conscious emotions[58]
9. Self-Efficacy for Exercise Scale (SEE)[59]
10. Appearance, body fat, strength, activity, endurance, and global physical subscales of the Physical Self Description Questionnaire - Short Form (PSDQ-S)[60]
11. Pittsburgh Sleep Quality Index (PSQI)[61]

Participants will also meet with a blinded outcomes assessor to complete the Hamilton Depression Rating Scale – 17-Item (HAM-D-17).[38]

The participants will undergo three functional physical tests:

1. Grip Strength[62]
2. Sit to Stand Test[63]
3. Push Up Test[64]

In addition, participants will complete the Concomitant Medication Record (CONMED).

Participants will obtain the Oura Ring[1] and a member of the research team will assist in the process of setting up the Oura Ring[1] for usage.

It is important to note that participants will obtain the Oura Ring[1] during the in-person baseline visit and will begin using it immediately, approximately 3 weeks prior to the start of the intervention period, to establish participants' baseline parameters. Participants will consistently use the Oura Ring[1] for a total of approximately 13 weeks (i.e., during the ~3 weeks of baseline, during the 4 weeks of the intervention period, and during the 6-weeks of follow-up).

During the 3-week buffer period for Oura Ring[1] baseline data collection, participants will also complete the following scales on a daily basis:

1. Patient Health Questionnaire - 2 item (PHQ-2; modified for daily administration)[54]
2. Generalized Anxiety Disorder Scale - 2 item (GAD-2; modified for daily administration)[55]
3. Measure of minutes of PA engaged in in the past day

   As with the Oura Ring[1] data collection, completion of the daily scales will continue throughout the study for a total of approximately 13 weeks (i.e., during the ~3 weeks of baseline, during the 4 weeks of the intervention period, and during the 6-weeks of follow-up).

Finally, the participant will be randomized into the PA group or TAU group as per Section 2.10. Depending on the outcome of the randomization, participants in the PA group will schedule their five sessions with their assigned trainer (see Section 2.12.1.3).

2.12.1.4 PA Intervention

Participants in the PA group will undergo a 4-week remotely delivered one-on-one individualized PA programme (for more details, see Section 2.4.1). They will meet with their trainers a total of five times. The first session will take place during the 3-week Oura Ring baseline period and will consist of psychoeducation and an introduction to the program. Participants will also undertake goal setting and activity planning. Following this session, the trainer will put together an individualized plan. During the intervention period, participants will engage in PA once weekly (for 4 weeks) during a supervised PA session with their trainer. They will also be instructed to independently engage in PA (outside of their 1-hour session with the trainer) for 120 minutes at

50-65% of their maximum work capacity. This can be done by engaging in PA between 3-5 times per week.

To ensure participants are well equipped to follow the PA program, they will be required to meet on a weekly basis with their assigned program trainer via Zoom, which will involve 30 minutes of behavioral change coaching and 30 minutes of the PA program designed by the program trainer.

At the beginning of each PA session, program trainers will ask participants how many minutes of PA they engaged in during the week (excluding the supervised PA session) and the type of activities they engaged in (prompts to include *with who*, *what*, and *where [e.g., indoors vs. outdoors]*).

Immediately after each supervised exercise session, program trainers will record participants' responses to the following scales:

- Feelings Scale[46]
- Felt Arousal Scale[47]
- Borg Rating of Perceived Exertion Category-Ratio Scale (BORG-CR-10)[44]
- Concomitant medication record (CONMED)
- Adverse event log (AE)
- Satisfaction with session rating

After the intervention period is complete, participants in the PA group will be asked to continue with their PA independently during the 6-week follow up period.

For the TAU group, a weekly call with a member of the study staff will be scheduled in lieu of the PA sessions to complete the CONMED and AE log. The TAU group will be given a handout with the Canadian 24-hour movement guidelines[42] and be told that they are encouraged to engage in PA.

2.12.5 Throughout the Study (Intervention and Follow-Up Periods)

Once a week during the intervention and follow-up periods, participants in both groups will complete the following scales via REDCap:

- World Health Organization-Five Well-Being Index (WHO-5; modified for weekly administration)[41]
- International Physical Activity Questionnaire - Long Form (IPAQ-L)[56]

On a daily basis, participants in both groups will complete the following scales via REDCap:

- Generalized Anxiety Disorder Scale - 2 item (GAD-2; modified for daily administration)[55]
- Patient Health Questionnaire - 2 item (PHQ-2; modified for daily administration)[54]
- Measure of minutes of PA engaged in in the past day

Participants in the control group will complete the same weekly and daily scales and will receive weekly phone calls to complete the CONMED and AE log (as mentioned above). Participants in the PA group will also receive weekly phone calls to complete the CONMED and AE log during the follow-up period.

All participants will be contacted to schedule a Zoom call at the end of the 4-week intervention period to complete the HAM-D-17[38] and two functional physical tests: Sit to Stand Test[63] and Push Up Test.[64] Participants will also complete the following scales via REDCap: PHQ-9,[39] GAD-7,[40] BAS-2,[57] body-related self-conscious emotions,[58] SEE,[59] the appearance, body fat, strength, activity, endurance, and global physical subscales of the PSDQ-S,[60] and the PSQI.[61]

In addition, during the intervention and follow-up periods, daily Oura Ring use will continue.

2.12.1.6 Week-6 Follow-Up Assessment (in-person)

The final follow-up assessments (Follow-up Week 6) will be completed in-person. The assessments will take approximately 2 hours to complete. First, participants will access the self-reported questionnaires through REDCap, including:

1. Patient Health Questionnaire - 9-item Scale (PHQ-9)[39]
2. Patient Health Questionnaire - 2 item (PHQ-2; modified for daily administration)[54]
3. Generalized Anxiety Disorder - 7-item Scale (GAD-7)[40]
4. Generalized Anxiety Disorder Scale - 2 item (GAD-2; modified for daily administration)[55]
5. World Health Organization-Five Well-Being Index (WHO-5; modified for weekly administration)[41]
6. International Physical Activity Questionnaire - Long Form (IPAQ-L)[56]
7. Body Appreciation Scale-2 (BAS-2)[57]
8. Body-related self-conscious emotions[58]
9. Self-Efficacy for Exercise Scale (SEE)[59]
10. Appearance, body fat, strength, activity, endurance, and global physical subscales of the Physical Self Description Questionnaire - Short Form (PSDQ-S)[60]
11. Pittsburgh Sleep Quality Index (PSQI)[61]

Participants will also meet with a blinded outcomes assessor to complete the Hamilton Depression Rating Scale – 17-Item (HAM-D-17).[38]

The participants will undergo three functional physical tests:
1. Grip Strength[62]
2. Sit to Stand Test[63]

3. Push Up Test[64]

In addition, participants will complete the following questionnaires:
1. Concomitant Medication Record (CONMED)
2. Adverse event log (AE)
3. Exit Survey (PA group only)

At this visit, the participant will be compensated the total amount for the study ($25 x 2 [in-person baseline and exit visits] + $50 [if participating in the optional semi-structured interview]). The participant will also return the Oura Ring and its charger during this visit.

2.12.1.7 Qualitative Interviews

During the 6-weeks following the PA intervention, semi-structured interviews will be scheduled with the participants in the PA group. The interviews will be conducted by a member of the study staff via Zoom and take approximately 45-60 minutes to complete.

Participants will also have the opportunity to participate in an optional semi-structured interview as part of a narrative study. The target sample size will be 7-10 participants. The interview will focus on collecting a story from participants about their relationship with PA and how they believe this relationship has intertwined with their depression. We will also ask participants to complete a timeline mapping exercise to recount the trajectory of their experiences with depression and PA. Time mapping is used to explore life events and trajectories across life stages and provides detailed information about transitions across one's life span. The interviews will take place at the participants convince any time during the study. Interviews are estimated to take between 60-90 minutes to complete. The audio of the interview will be recorded using Zoom and transcribed verbatim. Transcripts will be reviewed and compared to the Zoom recording to ensure verbatim transcription. Once completed, the transcripts will be sent to participants to review. Participants will be given 10 days to make any necessary edits and return the revised document to the researcher. The data analysis process will begin once the document has been reviewed and approved by the participant.

2.12.2 Retention

To promote participant retention and follow-up completion, we will provide participants with email reminders 24 hours before their scheduled appointments. Each research staff member will be easily available for the participants to contact via email or phone. We will be asking each participant to answer questions about the consent form to determine that the study process and the duration of participation are completely understood by all participants. The study team will work hard to form a professional relationship with the participant so that they feel comfortable and willing to discuss any relevant sensitive information. If a participant must reschedule a supervised PA session, it will be rescheduled to occur within the same week.

Participants will be compensated with $25 for each in-person visit (baseline and exit visits) to cover all travel expenses. Participants who agree to participate in the optional semi-structured interview for the narrative study will additionally be compensated with $50 for their time. They will be given the total study compensation at the exit visit when they return the Oura Ring. If participants withdraw from the study, they will receive the compensation that they are entitled to when they return the Oura Ring to the study team.

2.13 Data Analysis

We are primarily interested in generating essential data on potential feasibility and acceptability issues pertaining to a trial of a remotely-delivered, one-on-one, individualized PA programme vs. TAU in participants with TRD. We will assess feasibility and acceptability using descriptive statistics with counts and proportions for categorical data and means and standard deviations or median and interquartile range, as appropriate, for continuous data. The proportion lost to follow-up will be estimated along with a 95% confidence interval. The upper 95% confidence limit should not exceed 20% to proceed. The proportion compliant with the protocol, including treatment compliance as well as study completion and complete data on clinical outcomes (i.e., the per-protocol group) will be estimated with a 95% confidence interval. For validity, this should be fairly high so that the lower 95% confidence limit is > 80%. See Section 2.13.2 for additional acceptability analyses. We will estimate the means, standard deviations, and the correlations of HAM-D-17[38] and its changes over the study duration and over the follow-up. Also, we will estimate means, standard deviations and pre-post correlations of GAD-2,[55] PHQ-2,[54] GAD-7,[40] WHO-5,[41] and PHQ-9.[39] Initial analyses will summarize socio-demographic and clinical characteristics by treatment group. Comparative analyses will not be performed on clinical outcomes. Instead, means, standard deviations, and pre-post correlations will be estimated for continuous outcomes while proportions will be estimated for binary outcomes. One of the purposes of the vanguard phase is to measure outcomes for use on refining final sample size estimates but retain the ability to use the data collected in the final trial.

2.13.1 Oura Ring Activity Analysis Plan

Passive data from the wearable device will be analyzed using data-driven techniques to monitor a participant's activity in naturalistic environments. We will use MATLAB (MathWorks® Ltd) and Python platforms to conduct the statistical analysis.

2.13.2 Qualitative Data Analysis

Reflexive thematic analysis will be used for all qualitative data. The aim of reflexive thematic analysis is to focus on examining, pinpointing, and discovering patterns and identifying themes within the data set.[51,52] Reflexive thematic analysis is used when the existing body of knowledge on a topic is limited, as is the body of knowledge concerning PA interventions in TRD and how TRD impacts PA engagement across one's life. Reflexive thematic analysis means that the analysis

will center around researcher subjectivity, and a natural and recursive coding process in which reflection and iterative engagement with the data are valued.[52] Research grade qualitative analysis tools (NVivo 12 or Dedoose) will be used to organize and manage the data from the verbatim transcripts.[65,66]

2.14 Data Monitoring

2.14.1 Formal committee

Given that this is a feasibility trial with a small sample size, no Data Safety Monitoring Committee will be formed.

2.14.2 Interim analysis

There will be no planned interim analysis for this feasibility trial.

2.15 Privacy, Security, and Data Confidentiality

The confidentiality of the data collected and the identity of the individuals participating in this study will be strictly maintained. All files pertaining to subjects in the study will be assigned a unique ID. Personal identifiable information (PII), such as name, address, telephone number, email address, and date-of-birth, will not be accessible to anyone who is not authorized in the study conduct. Only the study staff who needs to have direct communications with the participants are authorized to access the PII. Source documents will always be kept in a locked filing cabinet to limit access, and in the case of electronic source documents, files will be password-protected and saved in a secure server. However, our CRFs will not contain any personal health information. Only the unique ID will be recorded in the CRF, and if the subject name appears on any other document, it must be de-identified and replaced with the unique ID from the copy of the document retained in the Trial Master File or made available for audit.

Study findings stored on a computer will be stored in accordance with UHT data and privacy regulations. The study staff, Principal Investigator and co-investigators will have access to all source documents collected over the course of the study. Participants will be informed that representatives of other parties, Research Ethics Board (REB), or regulatory authorities may inspect their records to verify that all information collected and made available for inspection will be handled in the strictest confidence and in accordance with UHT data and privacy regulations. The investigator will maintain a personal subject identification list (unique ID with the corresponding participant names) to enable records to be identified and retrieved.

Data collected by the Oura Ring will be de-identified on the Oura platform. Each participant's account on Oura will be created using a dummy email address and password with an associated unique ID. This deidentification will protect participant's privacy. Subsequently, the data will be transferred to an external encrypted and password-protected hard drive. The IPP will maintain

Oura Ring data ownership for all participants. This allows the IPP to delete completed study data from Oura Cloud.

During data analysis, de-identified data will be provided to the analysts. This will be achieved using a two-zone approach. The two-zone approach divides study team members into two groups; identified and de-identified. As the names suggest, the identified group will have access to identified (i.e., demographic data) and de-identified information and thus is able to re-identify the data to provide data management, quality and data linkage functions (on active and passive data) whereas the de-identified group will only have access to the de-identified information. This will ensure that the data analysts of the team only have access to de-identified data but will not have access to any of the participant's identifiers to protect participant privacy.

2.15.1 Source documents

Source data is all information, original records of clinical findings, observations, or other activities in a clinical trial necessary for the reconstruction and evaluation of the trial. Source data are contained in source documents.

Source data include:

UHT REB Documents

- All REB correspondences are documented.
- The study staff is REB approved prior to performing any study procedures.
- AEs and deviations are reported to the REB as per current guidelines and stored appropriately.
- All versions of the REB protocols and ICFs are on file.

Informed Consent

- Ensure that participant identification is not recorded on the informed ICF (i.e., no participant ID).
- There is documentation that the participant is given a copy of the consent form.
- The participant and study representative signed and dated the consent form for themselves.
- The participant initialed and dated all appropriate pages on the ICF.

2.16 Protocol Deviations

All deviations from the protocol will be addressed in study participant source documents. The researcher will complete a Protocol Deviation Log using the participant code as the identifier. This form will collect information such as the date the deviation occurred, details of what the deviation consisted of, any corrective and preventative actions that were taken as a result of the deviation, and the date that the investigator and REB were notified. The investigator will review the information and initial once approved. A completed copy of the Protocol Deviation Form will be maintained in the regulatory file and in the participant's source document. Protocol deviations will

be sent to the REB according to their guidelines. The site Principal Investigator or study staff will be responsible for knowing and adhering to their REB requirements.

2.17 Responsibilities

The responsibilities designated to each member of the research team are documented on the Delegation of Authority Form. A delegated staff member who is properly trained will be responsible for the informed consent process, review for eligibility, questionnaire administration, data entry, PA programme administration, and e-CRF entries.

2.18 Safety/Harms

2.18.1 Safety of PA

There may be the possibility of injury while participating in PA. Serious inherent risks are rare but include cardiac events and musculoskeletal injuries. To reduce the risk of sustaining any injuries, the PAR-Q[7] will be completed at screening and the PA program will be completed under the supervision of a program trainer who is a certified PA trainer with a background in exercise and health psychology. Participants are not required to perform any exercise that is deemed uncomfortable and will be instructed to not continue to exercise if the participant experiences sharp pain, nausea, dizziness, or light-headedness. The PA program is individualized and tailored to the needs and capabilities of each participant which limits the risk of injury.

2.18.2 AE definitions

AE: An AE is any untoward medical occurrence in a study participant receiving the PA programme and which does not necessarily have a causal relationship with this treatment. An AE can therefore be any unfavorable and unintended sign (including an abnormal laboratory finding), symptom, or disease temporally associated with the use of an investigational medical product (IMP), whether or not related to the IMP.

2.18.3 Collection of AEs

The condition of the participant will be monitored throughout the study. At each visit, whether scheduled or unscheduled, AEs will be recorded using an Adverse Events Log. In addition, the Principal Investigator and/or the co-investigators will check the participant records for any documented event.

Any AE which occurs during the study will be noted in detail on the appropriate pages of the CRF. If the participant reports several signs or symptoms representing a single syndrome or diagnosis, the diagnosis should be recorded in the CRF. The investigator will grade the severity of all AEs (mild, moderate, or severe), the seriousness (non-serious or serious), and the likelihood that they were related to the PA programme (causality).

Diseases, signs and symptoms, and/or laboratory abnormalities already present before the first administration of the PA programme will not be considered AEs unless an exacerbation in intensity or frequency (worsening) occurs.

The investigator will provide detailed information about any abnormalities and about the nature of and reasons for any action taken as well as any other observations or comments that may be useful for the interpretation and understanding of an AE.

2.18.4 Severity of AEs
The intensity/severity of AEs will be graded as follows:

Mild: an AE, usually transient, which causes discomfort but does not interfere with the participant's routine activities.

Moderate: an AE which is sufficiently discomforting to interfere with the participant's routine activities.

Severe: an AE which is incapacitating and prevents the pursuit of the participant's routine activities.

The grading of an AE is up to the medical judgment of the Investigator and will be decided on a case-by-case basis.

2.18.5 Causality of AEs
All AEs will be assessed by a blinded Investigator as to whether they can be explained by the participant's underlying condition. If they are not explainable, then the Investigator will make a determination of the relationship of the AE with the IMP (i.e., the PA programme) as follows:

- **Probable:** reports including good reasons and sufficient documentation to assume a causal relationship, in the sense of plausible, conceivable, likely, but not necessarily highly probable. A reaction that follows a reasonable temporal sequence from administration of the IMP and that could not reasonably be explained by known characteristics of the participant's clinical state.
- **Possible:** reports containing sufficient information to accept the possibility of a causal relationship, in the sense of not impossible and not unlikely, although the connection is uncertain or doubtful, for example because of missing data or insufficient evidence. A reaction that follows a reasonable temporal sequence from administration of the IMP but that could readily have been produced by several other factors.

- **Unlikely:** reports not following a reasonable temporal sequence from administration of the IMP. An event which may have been produced by the participant's clinical state or by environmental factors or other therapies administered.
- **Not related (unrelated):** events for which sufficient information exists to conclude that the etiology is unrelated to the IMP.
- **Unclassified:** reports which for one reason or another are not yet assessable (e.g., because of outstanding information (can only be a temporary assessment)).

2.18.6 Outcome of AEs

The outcome of all reported AEs has to be documented as follows:

1. Recovered, resolved
2. Recovering, resolving
3. Not recovered, not resolved (by study completion visit)
4. Recovered, resolved with sequelae
5. Fatal
6. Unknown

2.18.7 Procedures to manage AEs

Action(s) taken: AEs requiring action or therapy must be treated with recognized standards of medical care to protect the health and well-being of the participant. Appropriate resuscitation equipment and medicines must be available to ensure the best possible treatment in an emergency.

The action taken by the Investigator must be documented:

1. General actions taken in the event of an AE
    a. None
    b. Medication or other therapy (e.g., physical) started
    c. Test performed
    d. Other (to be specified)
2. IMP-related actions taken in the event of an AE
    a. None
    b. Intervention interrupted
    c. Intervention discontinued

The investigator will follow up on each AE until it has resolved or until the medical condition of the participant has stabilized. Any relevant follow-up information will be reported to the Principal Investigator (sponsor).

2.18.8 Serious Adverse Events (SAEs)

An **SAE** is any untoward medical occurrence that at any dose:

- results in death;

- is life-threatening (see below);
- requires hospitalization or prolongation of existing hospitalization;
- results in persistent or significant disability/incapacity;
- is another important medical event.

NOTE: The term ‘life-threatening’ refers to an event in which the participant was, in the view of the reporting Investigator, at immediate risk of death at the time of the event. It does not refer to an event which may hypothetically have caused death had it been more severe.

In deciding whether an AE is serious, medical judgment will be exercised. Thus, important AEs that are not immediately life-threatening or do not result in death or hospitalization but may jeopardize the participant or may require intervention to prevent one of the other outcomes listed in the definitions above should also be considered serious.

2.18.9 SAE Reporting Timelines

Reporting to REB: The study investigator will report all SAEs that are unexpected AND there is a reasonable possibility that the SAE is related to the research study to the REB within 7 calendar days of the study team becoming aware of the event. All fatal or life-threatening SAEs that are unexpected AND there is a reasonable possibility that the SAE is related to the research study will be reported within 3 days. Follow-up reports of the SAE will be submitted to the REB whenever new relevant information regarding the SAE becomes available until the resolution of the SAE.

2.19 Auditing

For this feasibility trial there will be no pre-specified independent audit. Representatives of SMH-UHT, including the SMH-UHT REB, may look at the study records and at personal health information to verify that the information collected for the study is correct and to make sure the study is following proper laws and guidelines.

3. ETHICS AND DISSEMINATION

3.1 Ethical Standard

The Principal Investigator will ensure that this study is conducted in full conformity with the ethical principles that have their origins in the Declaration of Helsinki. These principles are outlined by the Tri-Council Policy Statement: Ethical Conduct for Research Involving Humans - TCPS2 (2018, https://ethics.gc.ca/eng/policy-politique_tcps2-eptc2_2018.html) and codified in the International Council of Harmonization – Good Clinical Practice (ICH-GCP) E6.

3.2 Research Ethics Board (REB) Approval

This study will be conducted in accordance with the ethical principles laid down in the Declaration of Helsinki, the protocol, ICH GCP guidelines, and applicable regulatory requirements.

Full written informed consent will be obtained prior to conducting any study activities. The study will be reviewed and approved by the REB before any study related procedures commence.

3.3 Protocol Amendments
Before any changes to the study are implemented, besides those to eliminate immediate hazards to study participants, an amendment to the study will be reviewed and approved by the REB.

3.4 Informed Consent Process
Participants will be recruited from the IPP at SMH-UHT and other UHT affiliated hospitals using advertisements such as study posters. The single study centre will be the IPP at SMH-UHT.

Participants who appear to meet the eligibility criteria based on a preliminary REDCap screening survey will receive an email with a REDCap link to the ICF. The participants will be given sufficient time to read the ICF and ask any questions before deciding to participate. If participants agree to participate in the study, they will digitally sign/write their name on the REDCap ICF which will stand in place of a signature. An original ink signature will be collected at the in-person baseline visit.

3.5 Exclusion of Minorities and Children (Special Populations)
Non-English-speaking individuals are excluded because the ability to communicate study information, answer questions accurately and completely about the study, and obtain consent is necessary.

As part of the standard of care, pregnancy can limit PA capability. Therefore, pregnant females will not be considered for this study.

3.6 Confidentiality
Participant confidentiality is strictly held in trust by the participating investigators, their staff, and the research team. This confidentiality is extended to cover the clinical information relating to participants. All data will only be referenced by a numerical identifier code. Data will be stored on a password-protected computer, accessible to research personnel only. A key connecting names and code numbers will be kept in a locked cabinet, accessible only to the research personnel. Participants will not be identified in any report or publication about this study. See Section 2.15 Privacy, Security, and Data Confidentiality for more information on source documentation storage and security.

3.7 Study Discontinuation
If the study is discontinued, subjects who have completed or who are still enrolled in the study will be notified. Any new information gained during the study that might affect subjects' safety or

willingness to continue participation in the study will be communicated to participants by the study coordinator within 2 days after the Principal Investigator learns this information.

3.8 Declaration of Interests

Neither the Principal Investigator or Co-Investigators of this trial have any financial or competing interests to declare.

3.9 Ancillary and Post-Trial Care

No additional provisions will be made for post-trial care and routine clinical care will be provided by the participant's primary physician. If the participant suffers harm as a result of a study intervention, appropriate follow-up care will be provided as indicated.

3.10 Dissemination Policy

3.10.1 Trial Results

The results of this trial will be published in peer-reviewed journals and presented at scientific conferences/meetings.

3.10.2 Authorship

To be eligible for authorship on any resultant publications, all potential contributors must fulfill all criteria as set forth by the International Committee of Medical Journal Editors.

4. STUDY ADMINISTRATION

4.1 Key Contacts

Study Principal Investigator
Venkat Bhat, MD MSc FRCPC DABPN
St. Michael's Hospital and the University of Toronto
[redacted]

Study Co-Investigator
Catherine Sabiston, Ph.D
University of Toronto, Faculty of Kinesiology & Physical Education
[redacted]

4.2 Funding

This study will be supported by internal funding using existing sources.

4.3 Roles and Responsibilities

4.3.1 Sponsor and Funding

The study funder has no role in study design, collection, management, analysis and interpretation of data; writing of the report; and the decision to submit the report for publication.

5. REFERENCES

1. Oura Ring. Smart Ring for Fitness, Stress, Sleep & Health. https://ouraring.com

2. American Psychiatric Association. *Diagnostic and Statistical Manual of Mental Disorders (DSM-5).*; 2013.

3. Montgomery SA, Åsberg M. A New Depression Scale Designed to be Sensitive to Change. *Br J Psychiatry*. 1979;134(4):382-389. doi:10.1192/bjp.134.4.382

4. Sheehan DV, Lecrubier Y, Sheehan KH, et al. The Mini-International Neuropsychiatric Interview (M.I.N.I.): the development and validation of a structured diagnostic psychiatric interview for DSM-IV and ICD-10. *J Clin Psychiatry*. 1998;59 Suppl 20:22-33;quiz 34-57.

5. Dew RE. Adequacy of Antidepressant Treatment by Psychiatric Residents: The Antidepressant Treatment History Form as a Possible Assessment Tool. *Acad Psychiatry*. 2005;29(3):283-288. doi:10.1176/appi.ap.29.3.283

6. Kennedy SH, Lam RW, McIntyre RS, et al. Canadian Network for Mood and Anxiety Treatments (CANMAT) 2016 Clinical Guidelines for the Management of Adults with Major Depressive Disorder: Section 3. Pharmacological Treatments. *Can J Psychiatry*. 2016;61(9):540-560. doi:10.1177/0706743716659417

7. Warburton DER, Jamnik VK, Bredin SSD, et al. Executive Summary: The 2011 Physical Activity Readiness Questionnaire for Everyone (PAR-Q+) and the Electronic Physical Activity Readiness Medical Examination (ePARmed-X+). *Health Fit J Can*. Published online April 14, 2011:24-25 Pages. doi:10.14288/HFJC.V4I2.104

8. WHO. Depression: fact sheet. 2021. https://www.who.int/en/news-room/fact-sheets/detail/depression

9. Rush AJ, Trivedi MH, Wisniewski SR, et al. Acute and Longer-Term Outcomes in Depressed Outpatients Requiring One or Several Treatment Steps: A STAR*D Report. *Am J Psychiatry*. 2006;163(11):1905-1917. doi:10.1176/ajp.2006.163.11.1905

10. Cooney GM, Dwan K, Greig CA, et al. Exercise for depression. Cochrane Common Mental Disorders Group, ed. *Cochrane Database Syst Rev*. 2013;2013(9). doi:10.1002/14651858.CD004366.pub6

11. Peluso MAM, Andrade LHSGD. PHYSICAL ACTIVITY AND MENTAL HEALTH: THE ASSOCIATION BETWEEN EXERCISE AND MOOD. *Clinics*. 2005;60(1):61-70. doi:10.1590/S1807-59322005000100012

12. Conn VS. Depressive Symptom Outcomes of Physical Activity Interventions: Meta-analysis Findings. *Ann Behav Med*. 2010;39(2):128-138. doi:10.1007/s12160-010-9172-x

13. Dunn AL, Trivedi MH, O'Neal HA. Physical activity dose-response effects on outcomes of depression and anxiety. *Med Sci Sports Exerc*. 2001;33(6 Suppl):S587-597; discussion 609-610. doi:10.1097/00005768-200106001-00027

14. Craft LL, Landers DM. The Effect of Exercise on Clinical Depression and Depression Resulting from Mental Illness: A Meta-Analysis. *J Sport Exerc Psychol*. 1998;20(4):339-357. doi:10.1123/jsep.20.4.339

15. Wiles NJ, Haase AM, Lawlor DA, Ness A, Lewis G. Physical activity and depression in adolescents: cross-sectional findings from the ALSPAC cohort. *Soc Psychiatry Psychiatr Epidemiol*. 2012;47(7):1023-1033. doi:10.1007/s00127-011-0422-4

16. Goodwin RD. Association between physical activity and mental disorders among adults in the United States. *Prev Med*. 2003;36(6):698-703. doi:10.1016/S0091-7435(03)00042-2

17. Currier D, Lindner R, Spittal MJ, Cvetkovski S, Pirkis J, English DR. Physical activity and depression in men: Increased activity duration and intensity associated with lower likelihood of current depression. *J Affect Disord*. 2020;260:426-431. doi:10.1016/j.jad.2019.09.061

18. Wise LA, Adams-Campbell LL, Palmer JR, Rosenberg L. Leisure time physical activity in relation to depressive symptoms in the black women's health study. *Ann Behav Med*. 2006;32(1):68-76. doi:10.1207/s15324796abm3201_8

19. White RL, Babic MJ, Parker PD, Lubans DR, Astell-Burt T, Lonsdale C. Domain-Specific Physical Activity and Mental Health: A Meta-analysis. *Am J Prev Med*. 2017;52(5):653-666. doi:10.1016/j.amepre.2016.12.008

20. Motl RW, Konopack JF, McAuley E, Elavsky S, Jerome GJ, Marquez DX. Depressive Symptoms Among Older Adults: Long-Term Reduction After a Physical Activity Intervention. *J Behav Med*. 2005;28(4):385-394. doi:10.1007/s10865-005-9005-5

21. Guo F, Tian Y, Zhong F, Wu C, Cui Y, Huang C. Intensity of Physical Activity and Depressive Symptoms in College Students: Fitness Improvement Tactics in Youth (FITYou) Project. *Psychol Res Behav Manag*. 2020;Volume 13:787-796. doi:10.2147/PRBM.S267066

22. Teychenne M, White RL, Richards J, Schuch FB, Rosenbaum S, Bennie JA. Do we need physical activity guidelines for mental health: What does the evidence tell us? *Ment Health Phys Act*. 2020;18:100315. doi:10.1016/j.mhpa.2019.100315

23. Wang X, Cai Z dong, Jiang W ting, Fang Y yan, Sun W xin, Wang X. Systematic review and meta-analysis of the effects of exercise on depression in adolescents. *Child Adolesc Psychiatry Ment Health*. 2022;16(1):16. doi:10.1186/s13034-022-00453-2

24. Brellenthin AG, Crombie KM, Hillard CJ, Koltyn KF. Endocannabinoid and Mood Responses to Exercise in Adults with Varying Activity Levels. *Med Sci Sports Exerc*. 2017;49(8):1688-1696. doi:10.1249/MSS.0000000000001276

25. Leuenberger A. Endorphins, Exercise, and Addictions: A Review of Exercise Dependence. *IMPULSE*. Published online 2006.

26. Guerrera CS, Furneri G, Grasso M, et al. Antidepressant Drugs and Physical Activity: A Possible Synergism in the Treatment of Major Depression? *Front Psychol*. 2020;11:857. doi:10.3389/fpsyg.2020.00857

27. Lepore SJ. Expressive writing moderates the relation between intrusive thoughts and depressive symptoms. *J Pers Soc Psychol*. 1997;73(5):1030-1037. doi:10.1037/0022-3514.73.5.1030

28. Craft LL. Exercise and clinical depression: examining two psychological mechanisms. *Psychol Sport Exerc*. 2005;6(2):151-171. doi:10.1016/j.psychsport.2003.11.003

29. Dishman RK, Hales DP, Pfeiffer KA, et al. Physical self-concept and self-esteem mediate cross-sectional relations of physical activity and sport participation with depression symptoms among adolescent girls. *Health Psychol*. 2006;25(3):396-407. doi:10.1037/0278-6133.25.3.396

30. Greist JH, Klein MH, Eischens RR, Faris J, Gurman AS, Morgan WP. Running as treatment for depression. *Compr Psychiatry*. 1979;20(1):41-54. doi:10.1016/0010-440X(79)90058-0

31. Mota-Pereira J, Silverio J, Carvalho S, Ribeiro JC, Fonte D, Ramos J. Moderate exercise improves depression parameters in treatment-resistant patients with major depressive disorder. *J Psychiatr Res*. 2011;45(8):1005-1011. doi:10.1016/j.jpsychires.2011.02.005

32. Trivedi MH, Greer TL, Church TS, et al. Exercise as an Augmentation Treatment for Nonremitted Major Depressive Disorder: A Randomized, Parallel Dose Comparison. *J Clin Psychiatry*. 2011;72(05):677-684. doi:10.4088/JCP.10m06743

33. Firth J, Rosenbaum S, Stubbs B, Gorczynski P, Yung AR, Vancampfort D. Motivating factors and barriers towards exercise in severe mental illness: a systematic review and meta-analysis. *Psychol Med*. 2016;46(14):2869-2881. doi:10.1017/S0033291716001732

34. Lederman O, Suetani S, Stanton R, et al. Embedding exercise interventions as routine mental health care: implementation strategies in residential, inpatient and community settings. *Australas Psychiatry*. 2017;25(5):451-455. doi:10.1177/1039856217711054

35. Fibbins H, Lederman O, Rosenbaum S. Get Moving: Physical Activity and Exercise for Mental Health. In: *A Clinical Introduction to Psychosis*. Elsevier; 2020:493-510. doi:10.1016/B978-0-12-815012-2.00021-3

36. Adams SC, McMillan J, Salline K, et al. Comparing the reporting and conduct quality of exercise and pharmacological randomised controlled trials: a systematic review. *BMJ Open*. 2021;11(8):e048218. doi:10.1136/bmjopen-2020-048218

37. Glasgow RE, Vogt TM, Boles SM. Evaluating the public health impact of health promotion interventions: the RE-AIM framework. *Am J Public Health*. 1999;89(9):1322-1327. doi:10.2105/AJPH.89.9.1322

38. Hamilton M. A rating scale for depression. *J Neurol Neurosurg Psychiatry*. 1960;23(1):56-62. doi:10.1136/jnnp.23.1.56

39. Kroenke K, Spitzer RL, Williams JBW. The PHQ-9: Validity of a brief depression severity measure. *J Gen Intern Med*. 2001;16(9):606-613. doi:10.1046/j.1525-1497.2001.016009606.x

40. Spitzer RL, Kroenke K, Williams JBW, Löwe B. A Brief Measure for Assessing Generalized Anxiety Disorder: The GAD-7. *Arch Intern Med*. 2006;166(10):1092. doi:10.1001/archinte.166.10.1092

41. Topp CW, Østergaard SD, Søndergaard S, Bech P. The WHO-5 Well-Being Index: A Systematic Review of the Literature. *Psychother Psychosom*. 2015;84(3):167-176. doi:10.1159/000376585

42. Canadian Society for Exercise Physiology. Adults 18-64 – 24-Hour Movement Guidelines. Accessed April 29, 2024. https://csepguidelines.ca/guidelines/adults-18-64/

43. deJonge ML, Jain S, Faulkner GE, Sabiston CM. On campus physical activity programming for post-secondary student mental health: Examining effectiveness and acceptability. *Ment Health Phys Act*. 2021;20:100391. doi:10.1016/j.mhpa.2021.100391

44. Williams N. The Borg Rating of Perceived Exertion (RPE) scale. *Occup Med*. 2017;67(5):404-405. doi:10.1093/occmed/kqx063

45. Foster C, Porcari JP, Anderson J, et al. The Talk Test as a Marker of Exercise Training Intensity. *J Cardiopulm Rehabil Prev*. 2008;28(1):24-30. doi:10.1097/01.HCR.0000311504.41775.78

46. Hardy CJ, Rejeski WJ. Not What, but How One Feels: The Measurement of Affect during Exercise. *J Sport Exerc Psychol*. 1989;11(3):304-317. doi:10.1123/jsep.11.3.304

47. Svebak S, Murgatroyd S. Metamotivational dominance: A multimethod validation of reversal theory constructs. *J Pers Soc Psychol*. 1985;48(1):107-116. doi:10.1037/0022-3514.48.1.107

48. Moshe I, Terhorst Y, Opoku Asare K, et al. Predicting Symptoms of Depression and Anxiety Using Smartphone and Wearable Data. *Front Psychiatry*. 2021;12:625247. doi:10.3389/fpsyt.2021.625247

49. Callaghan P, Khalil E, Morres I, Carter T. Pragmatic randomised controlled trial of preferred intensity exercise in women living with depression. *BMC Public Health*. 2011;11(1):465. doi:10.1186/1471-2458-11-465

50. Doose M, Ziegenbein M, Hoos O, et al. Self-selected intensity exercise in the treatment of major depression: A pragmatic RCT. *Int J Psychiatry Clin Pract*. 2015;19(4):266-275. doi:10.3109/13651501.2015.1082599

51. Braun V, Clarke V. Using thematic analysis in psychology. *Qual Res Psychol*. 2006;3(2):77-101. doi:10.1191/1478088706qp063oa

52. Braun V, Clarke V. Reflecting on reflexive thematic analysis. *Qual Res Sport Exerc Health*. 2019;11(4):589-597. doi:10.1080/2159676X.2019.1628806

53. Calendly. https://calendly.com

54. Kroenke K, Spitzer RL, Williams JBW. The Patient Health Questionnaire-2: Validity of a Two-Item Depression Screener. *Med Care*. 2003;41(11):1284-1292. doi:10.1097/01.MLR.0000093487.78664.3C

55. Plummer F, Manea L, Trepel D, McMillan D. Screening for anxiety disorders with the GAD-7 and GAD-2: a systematic review and diagnostic metaanalysis. *Gen Hosp Psychiatry*. 2016;39:24-31. doi:10.1016/j.genhosppsych.2015.11.005

56. Craig CL, Marshall AL, Sjöström M, et al. International Physical Activity Questionnaire: 12-Country Reliability and Validity. *Med Sci Sports Exerc*. 2003;35(8):1381-1395. doi:10.1249/01.MSS.0000078924.61453.FB

57. Tylka TL, Wood-Barcalow NL. The Body Appreciation Scale-2: Item refinement and psychometric evaluation. *Body Image*. 2015;12:53-67. doi:10.1016/j.bodyim.2014.09.006

58. Sabiston CM, Doré I, Lucibello KM, et al. Body image self-conscious emotions get worse throughout adolescence and relate to physical activity behavior in girls and boys. *Soc Sci Med*. 2022;315:115543. doi:10.1016/j.socscimed.2022.115543

59. Resnick B, Jenkins LS. Testing the Reliability and Validity of the Self-Efficacy for Exercise Scale. *Nurs Res*. 2000;49(3):154-159. doi:10.1097/00006199-200005000-00007

60. Marsh HW, Martin AJ, Jackson S. Introducing a Short Version of the Physical Self Description Questionnaire: New Strategies, Short-Form Evaluative Criteria, and Applications of Factor Analyses. *J Sport Exerc Psychol*. 2010;32(4):438-482. doi:10.1123/jsep.32.4.438

61. Buysse DJ, Reynolds CF, Monk TH, Berman SR, Kupfer DJ. The Pittsburgh sleep quality index: A new instrument for psychiatric practice and research. *Psychiatry Res*. 1989;28(2):193-213. doi:10.1016/0165-1781(89)90047-4

62. Hamilton A, Balnave R, Adams R. Grip Strength Testing Reliability. *J Hand Ther*. 1994;7(3):163-170. doi:10.1016/S0894-1130(12)80058-5

63. Bohannon RW. Sit-to-Stand Test for Measuring Performance of Lower Extremity Muscles. *Percept Mot Skills*. 1995;80(1):163-166. doi:10.2466/pms.1995.80.1.163

64. Baumgartner TA, Oh S, Chung H, Hales D. Objectivity, Reliability, and Validity for a Revised Push-Up Test Protocol. *Meas Phys Educ Exerc Sci*. 2002;6(4):225-242. doi:10.1207/S15327841MPEE0604_2

65. Lumivero. NVivo. Published online 2023. https://lumivero.com/products/nvivo/

66. Dedoose. https://www.dedoose.com
