# Supplementary material for: Improving adherence to physical activity in treatment-resistant depression: Protocol for a pilot randomized controlled trial of a remotely delivered program
Source: PLoS One. 2025 Sep 2;20(9):e0330848. doi: 10.1371/journal.pone.0330848 (PMC12404371; doi:10.1371/journal.pone.0330848)
Supplement: S4 File — (PDF) [file pone.0330848.s004.pdf]

Letter of Information and Consent to Participate in a Research Study

Before agreeing to take part in this research study, it is important that you read the information in this research consent form. It includes details we think you need to know in order to decide if you wish to take part in the study. If you have any questions, ask a study doctor or study staff.

**Study Title:**	Remotely Delivered Physical Activity Programme for Treatment-Resistant Depression: A Pilot Randomized Controlled Trial
**Short Title:**	Physical Activity Programme for TRD
**REB Study Number:**	23-069
**Sponsor Investigator/Lead Investigator:**	Venkat Bhat, MD MSc FRCPC DABPN<br>St. Michael's Hospital and the University of Toronto<br>[redacted]
**Co-Investigators:**	Catherine Sabiston, PhD<br>University of Toronto, Faculty of Kinesiology & Physical Education<br>[redacted]
**Study Coordinator:**	Vanessa Tassone<br>Department of Psychiatry, St. Michael's Hospital<br>[redacted]
**Additional Study Personnel:**	Danika Quesnel<br>Department of Psychiatry, St. Michael's Hospital<br>[redacted]<br><br>Alyssa Teixeira<br>Department of Psychiatry, St. Michael's Hospital<br>[redacted]<br><br>Kevin Xiao<br>Department of Psychiatry, St. Michael's Hospital<br>[redacted]<br><br>Elyse Gorrell<br>Department of Psychiatry, St. Michael's Hospital<br>[redacted]<br><br>Monica Antunes<br>Department of Psychiatry, St. Michael's Hospital<br>[redacted]

Karisa Parkington
Department of Psychiatry, St. Michael's Hospital
[redacted]

Emma Li
Department of Psychiatry, St. Michael's Hospital
[redacted]

Fatemeh Gholamali Nezhad
Department of Psychiatry, St. Michael's Hospital
[redacted]

Gyu Hee (Sarah) Lee
Department of Psychiatry, St. Michael's Hospital
[redacted]

Josh Martin
Department of Psychiatry, St. Michael's Hospital
[redacted]

Alice Rueda
Department of Psychiatry, St. Michael's Hospital
[redacted]

CONFLICT OF INTEREST STATEMENT

The researchers are using internal funding from existing sources for this study and receive no direct or personal benefit from any study activities.

There is a risk of conflict of interest if you had previous relationships with any of the study staff. If that is the case, you may become ineligible to participate in the study.

Participation in this study is voluntary – you do not have to participate and you can withdraw at any time.

INTRODUCTION

You are invited to consider participating in this research study because you are experiencing a major depressive episode (MDE), have failed to respond to at least two trials of antidepressant therapy in the current depressive episode, and may be eligible to participate in a clinical trial of an individually tailored physical activity (PA) intervention.

Please read this Consent Form carefully and ask your primary doctor and study team as many questions as you would like before deciding whether to participate in this study. You may also wish to discuss the study with others, such as a family member or a close friend. This consent form should inform you of what this research is about and what your participation will involve. If you would like more details about any information that is included or not included here, please ask the research team via email or phone.

Please take your time in making your decision. Taking part in this study is voluntary. Deciding not to take part or deciding to leave the study later will not result in any penalty or affect your current or future health care.

You should be aware that it is possible that the St. Michael's Hospital, Unity Health Toronto study investigator(s) will also be your treating doctor(s).

If you choose to participate in this study, you will need to sign this Letter of Information and Consent form. You should not sign this form unless you are confident that you understand the information.

BACKGROUND AND PURPOSE OF THE STUDY

PA has been associated with improvements in mood and mental health in both clinical and non-clinical populations. Recent research suggests that low doses and enjoyability of PA underlie its' protective effect on mental health and well-being. As a result, our objective is to explore the impact of a supervised and personalized PA intervention program on depressive symptoms in participants with treatment resistant depression (TRD).

The purpose of the study is to assess the feasibility and acceptability of a trial delivering a 4-week remote, individualized, one-on-one PA program in addition to treatment as usual (TAU) in participants with TRD. At this time, we aim to conduct a pilot study with 30 participants to learn whether it will be possible to plan for a larger study in the future.

We will monitor the efficacy of the PA program on participants' depressive symptoms, anxiety symptoms, and quality of life to evaluate if it is effective at treating TRD.

STUDY DESIGN

This study is a randomized controlled trial (RCT), which means that participants will be randomly allocated into either an experimental group or a control group. Randomized clinical trials are studies where participants are randomly assigned to one of two groups. Neither you nor the research team can choose the group you will be assigned to. Participants in the experimental group will receive the remote PA intervention (4-week PA program and an Oura Ring) as an add-on to their usual treatment (TAU) whereas participants in the control group will receive TAU and an Oura Ring. Participants in the control group will be given a handout with the Canadian 24-hour movement guidelines and be told that they are encouraged to engage in PA, but will not receive the PA intervention. Twenty participants will be randomly assigned to the experimental group and the other 10 will be randomly assigned to the control group. At the end of their participation in the study, participants who were randomized to the control group will have access to at least one session with a PA trainer.

This study is not masked, which means that you will be aware of the group you are in. However, when it is time to analyze your data, the researchers will not know your information or which group you have been assigned to (control or experimental). Although you will know which intervention group you are assigned to, some members of the research team will not know which group you

belong to, as this information may unknowingly influence the views of the research staff in assessing your symptoms. Therefore, we ask you not to divulge your group assignment to any of the research members.

We are interested in the effects of PA on depressive symptoms, but also other factors such as anxiety and quality of life. As such, data will be collected digitally through REDCap, a web-based platform that has fillable questionnaires for you to answer. During study visits, self-report and study personnel-administered questionnaires will be completed to measure your symptoms.

Passive data will be collected through a wearable device (Oura Ring). More information on this platform can be found in the research activities section to follow.

If you are willing to participate in this research and are randomized into the intervention group, you will also be asked to participate in an online (Zoom) semi-structured interview with a member of the study staff who will ask about your experiences pertaining to the PA intervention at the end of the study. The interview will be approximately 45-60 minutes in length and will be audio recorded using Zoom. The recordings will be stored on an encrypted, password-protected hard drive. They will be transcribed verbatim and analyzed using reflexive thematic analysis to explore the participant's experiences in the PA program.

You will have the opportunity to participate in an additional optional online (Zoom) semi-structured interview which will explore your experience with PA across your life as part of a narrative study. This interview will be approximately 60-90 minutes in length and will also be audio recorded using Zoom.

STUDY DURATION

Overall, this study will run for about 17 months. However, your involvement in the study will last for about 13 weeks. This includes approximately 3 weeks for baseline parameter establishment, 4 weeks of the intervention period, and a 6-week follow-up period.

PARTICIPANT POPULATION AND STUDY ENROLMENT

This study will include 30 participants aged 18-65 (inclusive) who are diagnosed with TRD and are experiencing a MDE. A subset of 7-10 participants will be recruited for the narrative study.

Eligible individuals from the Interventional Psychiatry Program at St. Michael's Hospital will be recruited if they meet the eligibility criteria. Advertisements (e.g., flyers) will be posted in Unity Health Toronto affiliated hospitals for recruitment. Social media posts will also facilitate recruitment.

After indicating interest to a member of the study staff, participants will complete a pre-screening questionnaire via REDCap. Participants who appear eligible following the questionnaire will receive an email with the informed consent form, demographics form, medical/smoking/family history form, and Physical Activity Readiness Questionnaire (PAR-Q) to be signed/completed via REDCap. Please note that digitally signing/writing your name on the REDCap consent form

stands in place of a signature. You will also be prompted to schedule time for a Zoom visit to complete screening procedures. If eligibility is confirmed, an in-person baseline visit will be scheduled. You will pick up the wearable device at this visit.

Participants must have a physician (family doctor or psychiatrist) during their enrolment and participation in this study. The investigator will inform the participant's most responsible physician about their participation in the study.

Calendly will be used in this study to automate scheduling. Calendly works by providing you with time slots that you can select to schedule your visits; these time slots are selected and monitored by us.

To use this service, we will send you a link with the available time slots you can select to book your appointment, then, if you choose to use Calendly, you must add your first and last name and preferred email address. However, please note that Calendly will also collect other logging information for their own use. Some data collected may include the web address you are coming from or are going to, your device model, operating system, browser type, unique device identifier, IP address, mobile network carrier, and time zone or approximate location. To reduce collection of these log data by Calendly, please turn off non-necessary cookies.

By default, Calendly has all four types of cookies enabled. These cookies include:

Strictly necessary cookies: These cookies are necessary for the website to function and cannot be switched off. They are usually only set in response to actions made by you, which amount to a request for services, such as setting your privacy preferences, logging in, or filling in forms. You can set your browser to block or alert you about these cookies, but some parts of the site will then not work. These cookies do not store any personally identifiable information.

Performance Cookies: These cookies allow Calendly to count visits and traffic sources so they can measure and improve the performance of the site. They help Calendly know which pages are the most and least popular and see how visitors move around the site. All information these cookies collect is aggregated and therefore anonymous. If you do not allow these cookies, Calendly will not know when you have visited the site and will not be able to monitor its performance.

Functional Cookies: These cookies enable the website to provide enhanced functionality and personalization. They may be set by Calendly or by third party providers whose services have been added to the webpages. If you do not allow these cookies, some or all of these services may not function properly.

Targeting Cookies: These cookies may be set through Calendly by advertising partners. They may be used by those companies to build a profile of your interests and show you relevant adverts on other sites. They do not store directly personal information, but are based on

uniquely identifying your browser and internet device. If you do not allow these cookies, you will experience less targeted advertising.

IMPORTANT: You only need the Strictly necessary cookies to book your appointments with us. You DO NOT NEED the last three types of cookies in order to schedule with us. Therefore, you can unselect them to avoid sharing your personal information with Calendly's third party vendors and advertisers, and prevent them from identifying where you are.

If you do not feel comfortable having this information collected from Calendly, you can manually book your appointment with us via email.

Calendly is a US company with their server hosted by third party vendors in the US. Please see the list of Sub-processors that are contracted by Calendly to provide the services. You can find the link to the sub-processor list here: https://help.calendly.com/hc/en-us/articles/360047345493-Calendly-sub-processors-GDPR-CCPA-?_ga=2.78371863.281537918.1676301792-658059361.1664198325

In order to maintain privacy of your data, Calendly added secure measure to their service with data encryption. All connections from the browser to the Calendly platform are encrypted in transit using TLS SHA-256 with RSA Encryption. All data is encrypted when written to disk. Calendly user passwords are stored as salted password hashes. For more information, please visit Calendly's privacy and security statement at https://help.calendly.com/hc/en-us/articles/223146967-Your-privacy-and-security

Once the study is finished, we will request Calendly to delete our account and all information that can be removed. To obtain more information about Calendly's privacy, please go to https://calendly.com/privacy.

DESCRIPTION OF RESEARCH ACTIVITIES

Study Intervention

Physical Activity Program

If you decide to participate in this study, you may be randomly assigned to the experimental group where you will engage in a 4-week remotely delivered one-on-one individualized PA program. This will involve weekly sessions with a program trainer. You will first meet the program trainer prior to the PA intervention for an introduction session (approximately 1.5 hours). During this session, you will learn more about the PA program and getting started. You will also discuss your goals, needs, equipment access and previous PA experience with your assigned program trainer. This information will allow them to develop an individualized and tailored PA program that will start on your second session with the trainer and continue for 4 weeks.

During the intervention period, you will meet with the program trainer once a week virtually over Zoom for approximately 1 hour and 20 minutes. During this time, 30 minutes will be spent

engaging in behavior change coaching to help you set weekly goals, assess your goal progression, develop action plans, etc. The following 30 minutes will involve you engaging in a structured PA program that is tailored to your needs and goals. The PA program involves a warm up consisting of 5-7 minutes of light to moderate intensity cardiorespiratory and endurance activities, a conditioning component consisting of 20 minutes of individualized aerobic or resistance exercises and 3-5 minute cool down/stretch. The remaining 20 minutes will be dedicated to questionnaire completion. After each supervised session, you will also be asked to complete brief homework sections from a workbook used to guide the PA intervention and rate how satisfied you were with delivery of the session.

Each week, you will also be instructed to engage in PA for 150 minutes in total (including the 30-minute supervised PA session) at 50-65% of your maximum work capacity. It is important to note that you are only engaging in PA with the program trainer once a week for 30 minutes. The remaining 120 minutes of PA will be completed by you independently, on your own time, according to the tailored PA program developed by your program trainer. This can be done by engaging in PA between 3-5 times per week.

Prior to the start of each PA session, program trainers will ask you how many minutes of PA you engaged in during the week (excluding the supervised session) as well as the type of activities you engaged in. This information will also be cross-referenced with the data collected by the Oura App which will be explained in further detail below. After the 4-week supervised PA program is complete, you will be asked to independently continue with PA for 6 weeks.

RESEARCH TESTS AND PROCEDURES

In order for us to gain a better understanding of the severity of your symptoms, you will be asked to complete a few questionnaires throughout your involvement in the study using REDCap. REDCap is a software tool that helps researchers collect and manage data for their studies. Researchers can create online surveys using REDCap, which participants can fill out using their computers or mobile devices. As such, you will be sent a link to complete these REDCap surveys online. Not all surveys or study personnel-administered scales will be conducted every day, and a schedule with the approximate duration will be provided in the next section. Some of these surveys/questionnaires will be filled out on your own while others will be done with a member of the study team. Please note that the assessments marked with an asterisk (*) are semi-structured interviews or physical fitness tests conducted by the study team.

Research Questionnaires and Surveys

- Mini International Neuropsychiatric Interview (MINI)*
- Antidepressant Treatment History Form (ATHF)
- Montgomery-Åsberg Depression Rating Scale (MADRS)*
- 17-item Hamilton Depression Rating Scale (HAM-D-17)*
- Concomitant Medication Record (CONMED)
- Adverse Event Log (AE)
- Demographics Form
- Medical, Smoking and Family History form (MSF)

- Generalized Anxiety Disorder- 7-item scale (GAD-7)
- Generalized Anxiety Disorder - 2-item scale (GAD-2; modified for daily administration)
- Patient Health Questionnaire - 9-item scale (PHQ-9)
- Patient Health Questionnaire - 2-item scale (PHQ-2; modified for daily administration)
- International Physical Activity Questionnaire - Long form (IPAQ-L)
- Physical Activity Readiness Questionnaire (PAR-Q)
- Borg Rating of Perceived Exertion - Category Ratio Scale (BORG-CR-10)
- Feelings Scale (FS)
- Felt Arousal Scale (FAS)
- World Health Organization Well-Being Index-5 items (WHO-5; modified for weekly administration)
- Body Appreciation Scale-2 (BAS-2)
- Body-related self-conscious emotions
- Self-Efficacy for Exercise Scale (SEE)
- Select subscales of the Physical Self Description Questionnaire - Short Form (PSDQ-S)
- Pittsburgh Sleep Quality Index (PSQI)
- Grip Strength Test*
- Sit to Stand Test*
- Push Up Test*

During the interviews and questionnaires, some questions about your symptoms may seem personal or make you feel uncomfortable. You may take as long as you need to complete the questionnaires, and you may skip over any question or survey you do not wish to answer. If during the study you find that you are not comfortable doing the assessments, you are free to withdraw from participation. If you are having thoughts of hurting yourself or someone else, please talk to the study doctor or a research team member.

In the follow up period, participants randomized to the PA group will complete an exit survey to evaluate their experience of the PA program and will participate in a semi-structured interview with a research team member to further discuss the PA program.

<u>In addition to the clinical assessments, you will also use:</u>

Oura Ring – The Wearable Device

You will be required to use a wearable device (named "Oura Ring") throughout the duration of your participation in this study. This device will consist of a ring which has its own mobile application ("Oura" app) that needs to be downloaded onto your smartphone from Google Play (Android) or the App store (Apple). The study team will provide you with a dummy email address and password that you will use to login to your Oura account. In this way, data collection via the Oura Ring will be de-identified. Although the Oura app asks for your personal information when you first log in (e.g., sex, weight and height), **please DO NOT enter any personal information on the Oura app.**

The Oura Ring collects activity data and physiological signals (i.e., measurements related to body function such as heart rate or body temperature). The description of the passive data to be collected by Oura Ring is provided below as follows:

1. Sleep information: Oura Ring performs sleep analysis and stores a set of measurement parameters that summarize each period. The ring calculates the sleep period-specific parameters within four hours from the period end, but sleep analysis is always triggered when you open the application. These parameters include bedtime, total duration, awake, light, REM, and deep sleep durations, as well as heart rate, respiration rate, heart rate variability, and temperature changes.

2. Activity information: Activity summary contains daily activity summary values and detailed activity levels. Activity levels are expressed in metabolic equivalent of task minutes (MET mins). Oura tracks activity based on detected movement and presents the activity score based on duration of low, medium, and high activity levels. The movement is also used for step count and to estimate calories.

3. Readiness information: Readiness is interpreted from sleep, activity, resting heart rate, heart rate variability, recovery index and body temperature scores. A Readiness Score above 85% indicates that you're well recovered. A score below 70% usually means that an essential Readiness contributor, such as body temperature or previous night's sleep, falls outside your normal range, or clearly differs from recommended, science-based values.

You should know that Oura uses tools to monitor your activity on their websites. These tools will identify you by IP address, and will track which websites you visit and some of your activity on the website (such as how long you stay on the site). Oura may, at any time in the future, choose to use tools that also track and collect the information that you enter into a website, or that is collected by the ring and visible to you in their app; this may include data generated in this study. You can access the Oura Ring privacy policy here: https://ouraring.com/privacy-policy.

DESCRIPTION OF STUDY VISITS

Your participation in this study will last a total of approximately 13 weeks. You will be asked to come to St. Michael's Hospital for the baseline visit and the exit visit (more details below). The PA intervention and other study assessments will be completed remotely. You will continue taking your current medications during this study. It is important that your treatments remain unchanged one month before your screening visit and during your participation in the study.

Screening (approximately 1.5 hours, virtual)

If you agree to take part in this study, you will be screened to confirm your eligibility to participate in the study by the research team. During this visit:

1. We will collect information on your present medication.
2. You will be asked to complete an interview about depression called the Montgomery-Åsberg Depression Rating Scale (MADRS).
3. Complete the Mini International Neuropsychiatric Interview (MINI)
4. Complete the Antidepressant Treatment History Form (ATHF)

5. You will be asked to answer questions about the consent form to determine that the study process and the duration of participation are completely understood by all participants.

At this time, eligible participants will also be asked if they would like to participate in the optional semi-structured interview as part of the narrative study. You will be contacted by a member of the study team to schedule this interview to occur at your convenience.

Data obtained during screening will be retained and may be used for other research studies conducted at the Interventional Psychiatry Program at St. Michael's Hospital. If you have already undergone screening for another study at the Interventional Psychiatry Program, those data may be used to assess your eligibility for this study. Likewise, data collected during screening as part of this study may be used to assess your eligibility for other studies conducted at the Interventional Psychiatry Program.

Baseline Visit (approximately 2 hours, in-person)

If you are confirmed as eligible, you will complete baseline measures of the study's questionnaires including;

1. Patient Health Questionnaire - 9-item Scale (PHQ-9)
2. Patient Health Questionnaire - 2-item Scale (PHQ-2)
3. Generalized Anxiety Disorder - 7-item Scale (GAD-7)
4. Generalized Anxiety Disorder - 7-item Scale (GAD-2)
5. World Health Organization-Five Well-Being Index (WHO-5)
6. International Physical Activity Questionnaire - Long Form (IPAQ-L)
7. Body Appreciation Scale-2 (BAS-2)
8. Body-related self-conscious emotions
9. Self Efficacy for Exercise Scale (SEE)
10. Select subscales of the Physical Self Description Questionnaire - Short Form (PSDQ-S)
11. Pittsburgh Sleep Quality Index (PSQI)

Participants will also meet with a masked rater to complete the Hamilton Depression Rating Scale – 17-Item (HAM-D-17).

You will also undergo three short physical function tests:
1. Grip Strength Test
2. Sit to Stand Test
3. Push Up Test

During this visit, you will be randomly allocated to the PA group or the control group. If you are allocated to the PA group, you will schedule your five sessions with your PA program trainer. Individuals in the control group will have weekly check ins with a study team member.

At this visit, you will also be given the Oura Ring and a member of the research team will assist you in the process of setting it up for usage. You will begin using the Oura Ring immediately after the baseline visit. You will use it for approximately 3 weeks prior to the intervention period to establish baseline parameters. You will consistently use the Oura Ring throughout the study (i.e., during the 4-week intervention period and during the 6-week follow-up).

During the 3-week buffer period for Oura Ring data collection, you will also complete the following scales on a daily basis:

- Generalized Anxiety Disorder - 2-item scale (GAD-2)
- Patient Health Questionnaire - 2-item scale (PHQ-2)

These two daily scales will take approximately 5 minutes to complete. At this time, you will also be asked how many minutes of PA you engaged in in the past day. As with the Oura Ring data collection, completion of the daily scales will continue throughout the study (i.e., during the 4-week intervention period and during the 6-week follow-up).

Intervention Period (4 weeks)

The beginning of the intervention period starts on the third week of the study, even though you will have previously completed study visits (a Zoom screening visit, an in-person baseline visit, and one preliminary meeting with your program trainer if you are in the PA group). Participants randomized to the control group will be given a handout with the Canadian 24-hour movement guidelines and be told that they are encouraged to engage in PA. Participants randomized to the experimental group will engage in a 4-week individualized PA program that will involve a weekly supervised session with a program trainer delivered via Zoom. These sessions will last for approximately 1 hour and 20 minutes, with 30 minutes dedicated to behavior change coaching to help you set and assess weekly goals. The following 30 minutes will be dedicated to the structured PA program designed by the program trainer which will involve a combination of aerobic and resistance exercises. Although the exercises will differ from participant to participant, the overall program will be organized so that you start with 5-7 minutes of light to moderate intensity cardiorespiratory and endurance activities that will serve as the warm-up, followed by a 20-minute conditioning component made of aerobic and resistance exercises and finally a 3-5 minute cool down/stretch.

At the beginning of each PA session, program trainers will ask you how many minutes of PA you engaged in during the week (excluding the supervised PA session) and the type of activities you engaged in.

At the end of each supervised PA session, you will complete the following scales, which will take approximately 20 minutes:

- Borg Rating of Perceived Exertion - Category Ratio Scale (BORG-CR-10)
- Feelings Scale
- Felt Arousal Scale
- Concomitant medication record (CONMED)
- Adverse event log (AE)
- Satisfaction with session rating

Additional information will be collected on a daily basis through the Oura Ring and the following scales will be completed once a week:

- World Health Organization-Five Well-Being Index (WHO-5)
- International Physical Activity Questionnaire - Long Form (IPAQ-L)

The above scales will take approximately 10 minutes to complete.

It's important to note, that the following two scales will be completed on a daily basis:

- Generalized Anxiety Disorder - 2-item scale (GAD-2)
- Patient Health Questionnaire - 2-item scale (PHQ-2)

These two daily scales will take approximately 5 minutes to complete. At this time, you will also be asked how many minutes of PA you engaged in in the past day.

After the 4-week intervention period is complete, a member of the research team will contact participants via Zoom to complete the Hamilton Depression Rating Scale - 17-item scale (HAM-D-17) and two physical function tests (Sit to Stand Test and Push Up Test). Participants will also complete the following scales via REDCap:

- Patient Health Questionnaire - 9-item Scale (PHQ-9)
- Generalized Anxiety Disorder - 7-item Scale (GAD-7)
- Body Appreciation Scale-2 (BAS-2)
- Body-related self-conscious emotions
- Self-Efficacy for Exercise Scale (SEE)
- Select subscales of the Physical Self Description Questionnaire - Short Form (PSDQ-S)
- Pittsburgh Sleep Quality Index (PSQI)

The research member contacting participants will be masked which means they will not know if you are randomized to the control or PA group. As a result, it is important that you do not discuss the details of which group you are a part of to maintain masking. These clinical scales and physical function tests will take approximately 30-45 minutes to complete.

Participants in the control group will also complete the same daily and weekly scales (as mentioned above), will receive a weekly phone call to complete the CONMED and AE log, and will be contacted at the end of the 4-week intervention period to complete the HAM-D-17, Sit to Stand Test, and Push Up Test. They will also complete the following scales via REDCap:

- Patient Health Questionnaire - 9-item Scale (PHQ-9)
- Generalized Anxiety Disorder - 7-item Scale (GAD-7)
- Body Appreciation Scale-2 (BAS-2)
- Body-related self-conscious emotions
- Self-Efficacy for Exercise Scale (SEE)
- Select subscales of the Physical Self Description Questionnaire - Short Form (PSDQ-S)
- Pittsburgh Sleep Quality Index (PSQI)

<u>Follow-up Period (6 weeks)</u>

The follow-up period will begin at the end of the 4-week intervention period and will last 6 weeks. During this time, you will be asked to continue wearing the Oura Ring and to complete weekly/daily study measures as you were during the intervention period, regardless of which group you are in.

Those in the PA group will be encouraged to maintain their PA program independently. During the 6-week follow-up period, participants in the PA group will also participate in a semi-structured interview to discuss their experiences of the PA program and complete an exit survey. This will take approximately 45-60 minutes to complete.

A member of the research team will contact all participants weekly to complete the following:

- Concomitant medication record (CONMED)
- Adverse event log (AE)

Exit Visit (approximately 2 hours, in-person)

At the end of the 6-week follow up period, an in-person exit visit will be held where you will complete the self-reported questionnaires (including the PHQ-9, PHQ-2, GAD-7, GAD-2, BAS-2, body-related self-conscious emotions, SEE, select subscales of the PSDQ-S, and PSQI), as well as the HAM-D-17 with a masked member of the research team and the three physical function tests (Sit to Stand Test, Grip Strength Test, and Push Up Test). You will also return the Oura Ring and its charger during this visit. This visit will take approximately 2 hours to complete.

Please keep in mind that all visits may be shorter or longer in duration depending on individual circumstances.

Table of Study Visits and Research Activities

For your convenience, a table summarizing the study visits and outlining the research activities that will happen at each visit is included below. Please note that the baseline and exit visits will be in-person for all participants. The intervention period and remaining follow-up assessments will be delivered remotely.

Table 1: Study Procedures for Each Study Visit

	REDCap Screener/ Screening Visit	Baseline Visit	Buffer Period (3 weeks)	Intervention	Follow-up
Eligibility Screening	✓				
Oura Ring Pick Up/Return		✓ (pick up)			✓ (return; last follow-up)
Oura Ring Passive Wearable Data		✓	✓	✓	✓
Allocation		✓			
Scales, Questionnaires or Assessments					
Consent & Demographics	✓				

Adverse Event Log				✓	✓
Mini-international Neuropsychiatric Interview	✓				
Antidepressant Treatment History Form	✓				
Medical history, smoking history, family history	✓				
Physical Activity Readiness Questionnaire (PAR-Q)	✓				
International Physical Activity Questionnaire - Long form (IPAQ-L)		✓		✓	✓
Concomitant Medication Record	✓	✓		✓	✓
Montgomery-Åsberg Depression Rating Scale (MADRS)	✓				
Generalized Anxiety Disorder-7 (GAD-7)		✓		✓ (end of intervention)	✓ (last follow-up)
GAD-2		✓	✓	✓	✓
Patient Health Questionnaire-9 (PHQ-9)		✓		✓ (end of intervention)	✓ (last follow-up)
PHQ-2		✓	✓	✓	✓
World Health Organization Well-Being Index (WHO-5)		✓		✓	✓
Hamilton Depression Rating Scale - 17-item (HAM-D-17)		✓		✓ (end of intervention)	✓ (last follow-up)
Feelings Scale (FS)				✓	
Felt Arousal Scale (FAS)				✓	
Borg Rating of Perceived Exertion - Category Ratio Scale (BORG-CR-10)				✓	
Body Appreciation Scale-2 (BAS-2)		✓		✓ (end of intervention)	✓ (last follow-up)
Body-related self-conscious emotions		✓		✓ (end of intervention)	✓ (last follow-up)
Self-Efficacy for Exercise Scale (SEE)		✓		✓ (end of intervention)	✓ (last follow-up)
Physical Self Description Questionnaire - Short Form (PSDQ-S)		✓		✓ (end of intervention)	✓ (last follow-up)
Pittsburgh Sleep Quality Index (PSQI)		✓		✓ (end of intervention)	✓ (last follow-up)

Sit to Stand Test		✓		✓ (end of intervention)	✓ (last follow-up)
Grip Strength Test		✓			✓ (last follow-up)
Push Up Test		✓		✓ (end of intervention)	✓ (last follow-up)
Adapted MoveU.HappyU Workbook			✓	✓	
Exit Survey					✓ (last follow-up)
Semi-Structured Qualitative Interview	✓ (optional narrative interview any time during study)				✓

PARTICIPANT RESPONSIBILITIES

If you decide to take part in this study, it is important that you remember to:

- Attend the weekly Zoom sessions with your program trainer (note: if a supervised PA session must be rescheduled, it will be rescheduled to occur within the same week)
- Engage in PA for 150 minutes per week at 50-65% of your maximum work capacity if you are in the experimental group
- Complete weekly homework assignments
- Complete questionnaires on REDCap when instructed
- Continually use the Oura Ring
- Ask your study team about anything that worries you
- Tell study staff about any changes in your health
- Do not change your medications or treatments during the 4 weeks before the study, the 4 weeks of the intervention period, and the 6-week follow up period
- Tell study staff if you are considering any changes to your treatment, medications or doses
- Tell study staff if you have changed any of your treatment, medications or doses
- Tell study staff if you suspect you are pregnant
- Tell your study team if you change your mind about being in this study
- Tell your study team if you are considering enrolling in another study

Additionally, we will conduct weekly compliance calls wherein you will receive a phone call if Oura data is missing for 3 or more days in the past week or if REDCap data is missing. This phone call is designed to prevent any future missingness by helping you troubleshoot and answer any questions or concerns you may have.

POTENTIAL RISKS OR HARMS OF PARTICIPATING IN THIS STUDY

Physical Activity:

It is possible to sustain an injury while engaging in PA. Serious inherent risks are rare but include cardiac events and musculoskeletal injuries. To reduce the risk of sustaining any injuries, the Physical Activity Readiness Questionnaire (PAR-Q) will be completed at screening and the PA

program will be completed under the supervision of a program trainer who is a certified PA trainer with a background in exercise and health psychology. Participants are not required to perform any PA that is deemed uncomfortable and will be instructed to not continue the activity if the participant experiences sharp pain, nausea, dizziness or light-headedness. The PA program is individualized and tailored to the needs and capabilities of each participant which limits the risk of injury.

Wearable Device (Oura Ring):

If you agree to participate, you will be asked to use the wearable device and its app throughout the study. You may experience a burden or a negative reaction when using the wearable device and its app as you will be asked to wear the device whenever possible and to recharge it when necessary (approximately every 5-6 days). You may also experience negative feelings or reactions when accessing your data on the wearable app. If any of these things happen, you can pause or stop using the wearable device and wearable app, or you can also choose to withdraw from the study. If you choose to pause or stop your use of the wearable device and app, please inform a member of the study team. Additionally, you may have an allergic reaction while using the wearable device; in this case, you should stop using the device and contact your family physician. Please avoid handling batteries or working on devices with machinery that contain batteries while wearing your Oura Ring. In certain cases, where both the cathode and the anode of another battery touch the ring, there is a risk of a short circuit which is similar to standard metallic rings. This can result in a potentially dangerous shock. Most importantly, if you experience a negative reaction or distress, you may contact the study staff, as they are available to answer any questions you might have regarding the study, the wearable device and its app, or where to seek assistance if needed. If you need immediate assistance, please call 911 and go to the Emergency Room closest to you.

Clinical Assessments:

There is a risk that you may feel uncomfortable discussing some of the topics in the clinical assessment and completing some of the questionnaires. You can skip questions that you do not wish to answer, or you can pause or stop your participation in the study. If you have any questions or concerns while answering these questions, the study team is available to discuss your concerns and/or to refer you to appropriate resources.

POTENTIAL BENEFITS

There may not be any direct benefits to you for participation in this study. However, you may experience some relief of your depression symptoms and there is a possibility that your condition may improve faster or to a greater extent than it did when using prior antidepressant regimens. There will be no difference in your care if you choose to take part in the study or not. Your participation may allow the researchers to establish whether PA programs are feasible and safe procedures for those with depression. Further studies looking at the efficacy of PA programs will be needed to learn whether it can treat depression, which may be of benefit to future participants.

ALTERNATIVES TO PARTICIPATION

This study is not researching ways to provide you with medical treatment, so the alternative to taking part in this study is to not take part in the study. Whether you choose to take part in this study or not, you will receive the same standard and level of care at Unity Health Toronto.

PRIVACY AND CONFIDENTIALITY OF YOUR PERSONALLY IDENTIFYING INFORMATION AND STUDY DATA

This section describes how your personal identifying information and study data will be accessed, disclosed, and stored during this study. All people involved in this study are committed to respecting your privacy. Other than the individuals or groups described in this section, no people will have access to your personally identifying information without your consent, unless required by law.

Personally, identifying information is any information that could be used to identify you; this includes your name, date of birth, email address, and telephone number.

Study data is information that is generated by and/or collected for a study that has been stripped of personally identifying information.

PROTECTING YOUR PRIVACY

The study personnel will make every effort to keep your personally identifying information private and confidential in accordance with all applicable privacy legislation, including the Personal Health Information Protection Act (PHIPA) of Ontario.

Any information that is recorded for study purposes will be de-identified by using a unique study identification number instead of any of your personal identifiers. The Principal Investigator at St. Michael's Hospital is in control of the key that links your study number to you personally.

No personally identifying information will be allowed off-site in any form, unless required by law or as described in this consent form.

Data collected via Oura will be transferred from the Oura servers, which use Amazon Web Services in the European Union, to an external encrypted and password-protected hard drive. By signing this consent form, you agree to transfer ownership of the data collected from the Oura Ring for the study to the study team, such that the study team has the authority to delete the study data permanently from the Oura server. Participant data will be accessible from the Oura servers until study completion. Upon study completion or dropout, the study team will deactivate all Oura accounts and participant data will be permanently removed from the Oura server. Data collected via surveys completed through REDCap will be stored on the REDCap servers located at Unity Health Toronto. Demographic information, including personally identifying information will be stored on a Unity Health Toronto server.

Your research records, including personally identifying information, will be kept for 7 years after study completion at St. Michael's Hospital in a highly secure and confidential manner, as this is the length of time required by Unity Health Toronto's policy. The information will then be anonymized (any link to you will be destroyed) or completely destroyed.

USE OF EMAIL/TEXTING FOR RESEARCH

There are common risks of using email and/or texting to communicate:

- Information travels electronically and is not secure in the way a phone call or regular mail would be.
- If someone sees these emails and/or texts they may know that you are a participant in this study or see the health information included in the email and/or text.
- Emails and/or texts may be read or saved by your internet or phone provider (i.e., Rogers, your workplace, "free internet" providers).
- Copies of an email and/or text may continue to exist, even after efforts to delete the email and/or text have been made.
- There is always a chance with any unencrypted email and/or text, however remote, that it could be intercepted or manipulated.

Do not use email and/or text messaging for medical emergencies. If you require immediate help, call your clinic, or care provider, or seek emergency services.

PERSONALLY IDENTIFYING INFORMATION STORAGE AND RETENTION

All your personally identifying information will be collected on a database for analysis at St. Michael's Hospital. The personally identifying information will be stored on a secure internal hospital server and will be password protected so that it is only accessible to authorized research personnel.

Your personally identifying information will be kept by the Principal Investigator and Unity Health Toronto for 7 years after study completion at St. Michael's Hospital in a highly secure and confidential manner, as per hospital requirements. After 7 years, any documents with personally identifying information will be destroyed.

Original audio recordings, which may contain personally identifying information, will be stored on an encrypted, password-protected hard drive and will be destroyed after 7 years.

STUDY DATA STORAGE AND RETENTION

As a reminder, study data is information that is generated by or collected for a study that has been stripped of personally identifying information. The study data includes: data collected from REDCap questionnaires and case report forms, Oura Ring data, interview transcripts, and demographic information. Study data will be securely stored at Unity Health Toronto. Study data may also be transferred outside of Unity Health Toronto and shared with others for purposes related to the conduct of this study.

Study data may be kept indefinitely and may be used for other research or analyses by the study investigators and the study sponsor.

Individual level study data may also be made available to scientific journals, their reviewers, other researchers inside or outside of Unity Health Toronto, or the public.

STUDY RESULTS AND REGISTRATION

RESULTS

The results of this study may be presented at a scientific conference or published in a scientific journal. If you are interested in obtaining the results of this study, you can contact the study team. We estimate that the results of this study will be available in 2 years.

You will never be personally identified in any publication, report, or presentation that may come from this study. However, direct quotes from the semi-structured interviews may be used in publications resulting from the study.

REGISTRATION

A description of this clinical trial will be available on http://www.ClinicalTrials.gov. This website will not include information that can identify you. At most, the website will include a summary of the results. You can search this website at any time.

The registration number for this study is NCT06404320.

POTENTIAL COSTS AND REIMBURSEMENT

There are no costs to you for participation in this study. You will not be paid for your participation in this study.

You will be compensated up to $25 for the in-person baseline and exit visits, for a total of $50, to cover all travel expenses. We are not able to provide compensation for additional expenses such as food. If you agree to participate in the optional semi-structured interview for the narrative study, you will be compensated with an additional $50 for your time. You will receive the total compensation at the end of your involvement in the study, at the exit visit, upon returning the Oura Ring. No receipts will need to be provided. If you withdraw from this study before completing it, you will receive compensation for the parts of the study that you have completed.

If a discovery is made or a commercial product or method is derived from this study, it will be the property of the study sponsor and you will not be entitled to any financial benefits resulting from it.

COMPENSATION FOR INJURY

If you experience side effects from study interventions or study procedures, you should inform the study team as soon as possible. If you have any other concerns about safety or unexplained symptoms, contact us immediately.

If you are injured because of your participation in this study, medical care will be provided to you in the same manner as you would ordinarily obtain any other medical treatment. In no way does signing this form waive your legal rights nor release the study doctor(s), sponsor, or involved institution(s) from their legal and professional responsibilities.

PARTICIPATION IN THE STUDY

Your participation in this study is voluntary. If you choose not to participate, there will be no impact on the medical care received at, employment at, or other relationships with Unity Health Toronto now or in the future for you or your family.

WITHDRAWAL FROM THE STUDY

If you choose to take part in this study, you can change your mind without giving a reason, and you may withdraw from the study at any time without any effect on the care you or your family will receive at St Michael's Hospital. If at any time you choose to withdraw from this study, please contact a member of the study team.

Your participation in the study may be stopped without your consent for the following reasons:

- If continuation in the study appears to be harmful to you
- If it is discovered that you do not meet the eligibility requirements
- If you fail to adhere to the study's responsibilities

The study may be terminated by the investigators at any time for any reason.

CONTINUED COLLECTION AND USE OF YOUR DATA AFTER WITHDRAWAL

If you withdraw from the study, any data collected from you up to that time will still be used. No more data from you will be collected unless it is necessary to follow up on an adverse event that is not resolved at the time of your withdrawal. We may be required to retain the personally identifying information and study data that we have already collected until after the end of this study (described in the Privacy and Confidentiality section).

You will not have access to the study intervention after your involvement in the study.

NEW INFORMATION ABOUT THIS STUDY

We may make changes to the study. We may also learn new things about the study that you may need to know. Some of the new information or changes might affect your decision to take part in

the study. If so, you will be notified about the new or changed information in a timely manner and we will ask you if you consent to remain in the study. You may be asked to sign a new consent form at that time.

NEW INFORMATION ABOUT YOUR HEALTH (INCIDENTAL FINDINGS)

The tests or procedures that we conduct during this study might reveal medical information about you that is not part of the objectives of this study but may be relevant to your health. This type of medical information is called an incidental finding. Some incidental findings could be related to treatable conditions, or they could be related to factors that may affect your current or future health care. With your consent, we will communicate all medically actionable incidental findings to you.

RESEARCH ETHICS BOARD CONTACT

If you have any questions regarding your rights as a research participant, you may contact the Unity Health Toronto Research Ethics Board Office at:

Unity Health Toronto Research Ethics Board
Monday to Friday, 9:00 a.m. to 5:00 p.m.
[redacted]

Unity Health Toronto is a health network that includes Providence Healthcare, St. Joseph's Health Centre, and St. Michael's Hospital.

The Unity Health Toronto Research Ethics Board is made up of a group of scientists, medical staff, and individuals from other backgrounds (including law and ethics) as well as members from the community. The Board was established by Unity Health Toronto to review studies for their scientific and ethical merit. The Board pays special attention to the potential risks and benefits to the research participant, as well as the potential benefit to society.

STUDY CONTACTS

If you have questions about taking part in this study, or if you suffer a research-related injury, you can talk to the research team, or the person who oversees the study at this institution. That person is:

Venkat Bhat, MD, MSc, FRCPC, DABPN
St. Michael's Hospital and University of Toronto
[redacted]
(You will be asked to leave your phone number to be called)

Signature Pages: Documentation of Informed Consent

Study Title: Remotely Delivered Physical Activity Programme for Treatment-Resistant Depression: A Pilot Randomized Controlled Trial

Sponsor Investigator/Lead Investigator: Venkat Bhat, MD, MSc, FRCPC, DABPN
[redacted]

Participant Statement of Consent

The research study has been explained to me, and my questions have been answered to my satisfaction. I have been informed of the alternatives to participation in this study. I have the right not to participate and the right to withdraw without affecting the quality of medical care at St. Michael's Hospital for me and for other members of my family. As well, the potential harms and benefits (if any) of participating in this research study have been explained to me.

I have been told that I have not waived my legal rights nor released the investigators or involved institutions from their legal and professional responsibilities. I know that I may ask now, or in the future, any questions I have about the study. I have been told that records relating to me and my care will be kept confidential and that no information will be disclosed without my permission unless required by law. I am aware that my physician (family doctor or psychiatrist) will be notified about my participation in this study. I have been given sufficient time to read the above information.

I consent to participate. I have been told I will be given a signed copy of this consent form.

______________________	______________________	__________	__________
Participant name (print)	Participant signature	Date	Time

I would like to participate in the optional semi-structured interview as part of the narrative study.

___Yes ___No

I have explained to the above-named participant the nature and purpose, the potential benefits, and possible risks of participation in this study. All questions that have been raised about this study have been answered.

______________________	______________________	______________________	__________	__________
Name of person obtaining consent (print)	Position/Title of person obtaining consent (print)	Signature of person obtaining consent	Date	Time

Please initial one of the boxes below to indicate whether or not you want to be informed of all medically actionable incidental research findings.

	**YES**, I agree to be told about any medically actionable incidental findings.
	**NO**, I do not want to be told about medically actionable incidental findings.
